# PD‐L1 on Tumor‐Derived Extracellular Vesicles Induces CD8^+^ T Cell Terminal Exhaustion and Mediates Anti‐PD‐1 Resistance in Head and Neck Squamous Cell Carcinoma

**DOI:** 10.1002/advs.202516348

**Published:** 2025-11-05

**Authors:** Ruihua Fang, Bixue Huang, Yun Li, Zhimou Cai, Yicheng Deng, Xiaoqing Cen, Wei Sun, Jinhong Zhang, Zhenglin Xu, Wenbin Guo, Yi Lyu, Shiyun Luo, Zhesheng Chen, Kexing Lyu, Weiping Wen, Wenbin Lei

**Affiliations:** ^1^ Department of Otorhinolaryngology Head and Neck Surgery The First Affiliated Hospital Sun Yat‐Sen University Guangzhou Guangdong 510080 P. R. China; ^2^ Department of Pharmaceutical Sciences College of Pharmacy and Health Sciences Institute for Biotechnology St. John's University 8000 Utopia Parkway, Queens New York 11439 USA

**Keywords:** head and neck squamous cell carcinoma, immunotherapy resistance, PD‐L1, terminally exhausted T cells, tumor‐derived extracellular vesicles

## Abstract

Head and neck squamous cell carcinoma (HNSCC) demonstrates suboptimal responses to current immune checkpoint inhibitors (ICIs), with objective response rates (ORRs) of merely 15–20%. The molecular mechanisms underlying these low ORRs remain incompletely defined. Here, two functionally distinct CD8⁺ T cell subsets are identified within the tumor microenvironment: precursor exhausted T (Tex^prog^) cells and terminally exhausted T (Tex^term^) cells. Notably, although anti‐PD‐1 therapy reduced Tex^prog^ cell frequencies, it failed to reverse Tex^term^ cells. Elevated Tex^term^ cell infiltration correlated with advanced tumor–node–metastasis (TNM) staging and poor prognosis. Furthermore, non‐responders exhibited significantly higher baseline Tex^term^ proportions than responders before immunotherapy. Multivariate analysis established stromal Tex^term^ cell density as both an independent prognostic factor and predictor of ICIs resistance. Mechanistically, Tex^term^ cell infiltration strongly correlated with PD‐L1 on tumor‐derived extracellular vesicles (PD‐L1^+^EVs). Most importantly, it is demonstrated that PD‐L1^+^EVs drive Tex^term^ cell differentiation by upregulating the basic leucine zipper transcription factor, ATF‐like (BATF) in CD8⁺ T cells. Knocking out PD‐L1 on EVs reduced Tex^term^ cell infiltration and BATF expression. These findings elucidate an EV‐mediated immune evasion axis and reveal actionable targets to overcome immunotherapy resistance.

## Graphical abstract

Immunotherapy fails in head and neck squamous cell carcinoma (HNSCC) due to terminally exhausted T (Tex^term^) cells. Tumor‐derived PD‐L1^+^ extracellular vesicles promote CD8‐Tex^term^ cells differentiation by upregulating BATF. Stromal Tex^term^ density serves as a potent biomarker for predicting immunotherapy resistance, highlighting a novel EV‐mediated mechanism and potential target to overcome immunotherapy failure in HNSCC.

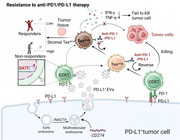

## 1 Introduction

Immune checkpoint inhibitors (ICIs), particularly anti‐PD‐1 (aPD‐1) agents, induce durable clinical responses across patients with solid malignancies.^[^ ^1^, ^2^, ^3^ ^]^ While aPD‐1 monotherapy achieves ≥40% objective response rates (ORRs) in advanced melanoma,^[^ ^4^ ^]^ renal cell carcinoma,^[^ ^5^ ^]^ endometrial cancer,^[^ ^6^ ^]^ non‐small cell lung cancers,^[^ ^7^ ^]^ and others, HNSCC exhibits profoundly attenuated efficacy—with ORRs languishing below 20%.^[^ ^8^, ^9^ ^]^ This profound disparity fundamentally emanates from the uniquely immunosuppressive tumor microenvironment (TME) inherent to HNSCC, characterized by high infiltration of immunosuppressive cells^[^ ^10^, ^11^ ^]^ and the functional exhaustion of CD8^+^ T cells.^[^ ^12^ ^]^ Despite extensive research into immunosuppressive cells yielding no therapeutic breakthroughs to meaningfully improve clinical outcomes, elucidating the molecular mechanisms of CD8^+^ T cell exhaustion and expanding functional subsets remain pivotal strategies for enhancing antitumor immunity.

Recently, single‐cell transcriptomic studies have demarcated a hierarchical CD8^+^ T cell exhaustion continuum comprising two functionally heterogeneous subsets: progenitor exhausted T (Tex^prog^, PD‐1^+^TCF‐1^+^TIM‐3^−^CD8^+^) cells and terminally exhausted (Tex^term^, PD‐1^+^TCF‐1^−^TIM‐3^+^CD8^+^) cells.^[^ ^13^, ^14^ ^]^ Tex^prog^ cells retain proliferative plasticity and constitute the primary reservoir responsive to ICIs through TCF‐1‐mediated self‐renewal. Conversely, Tex^term^ cells undergo epigenetic constriction of effector loci and mitochondrial metabolic collapse, culminating in irreversible hypofunction.^[^ ^15^, ^16^ ^]^ Upon ICIs administration, Tex^prog^ cells in the TME can be reactivated into effector CD8^+^ T cells, thereby restoring anti‐tumor immunity. In contrast, the irreversible dysfunction of Tex^term^ cells cripples antitumor immunity and drives immunotherapy failure. Research indicates that CD8^+^ exhaustion cells represent a developmental continuum, wherein the Tex^term^ population predominantly arises from the differentiation of Tex^prog^ cells.^[^ ^17^ ^]^ However, the regulatory mechanisms governing the multi‐step differentiation process from effector CD8^+^ T cells to Tex^term^ cells remain poorly understood.

In the present study, we identified high stromal infiltration of Tex^term^ cells in immunotherapy‐resistant HNSCC patients through an integrated analytical approach combining single‐cell RNA sequencing (scRNA‐seq), flow cytometry, and multiplex immunohistochemistry (mIHC). Furthermore, a strong positive correlation was observed between Tex^term^ cell abundance and PD‐L1 expression on tumor‐derived extracellular vesicles (PD‐L1^+^ EVs). To investigate how PD‐L1^+^ EVs drive Tex^term^ cell generation, we successfully established PD‐LI^KO^ EVs using CRISPR‐Cas9 technology, as verified in our prior studies.^[^ ^11^ ^]^ Using subcutaneous transplanted tumor and tail vein administration models in mice, we further confirmed that PD‐L1^+^ EVs drive the differentiation and generation of CD8^+^ T cells into Tex^term^ cells through a BATF‐mediated transcriptional program.

## 2 Results

### 2.1 Anti‐PD‐1 Treatment Cannot Reverse the Terminal Exhaustion of T Cells

**Figure 1:**
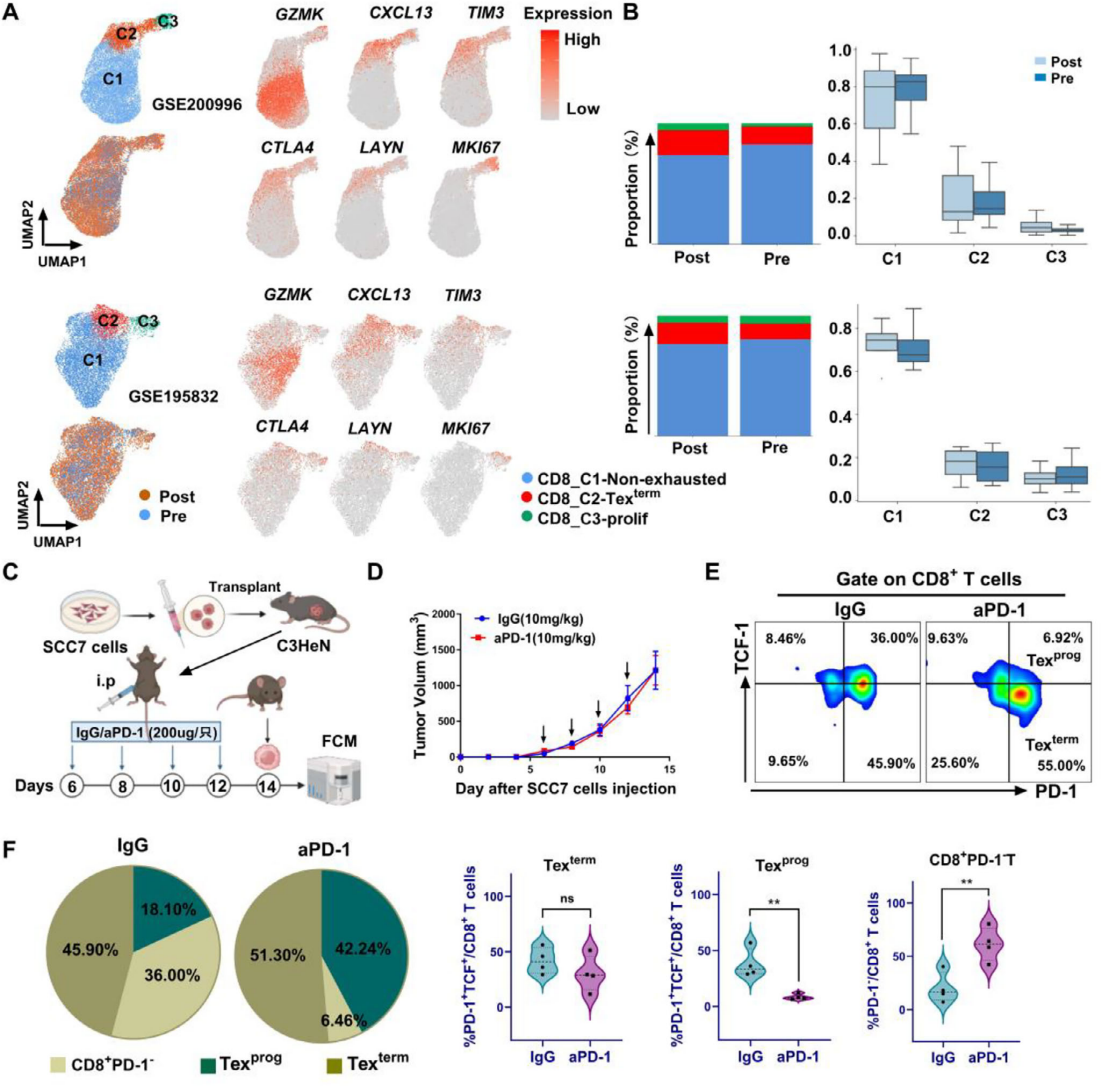
Differences in the number of tumor‐infiltrating exhausted T cells before and after aPD‐1 treatment in HNSCC. A) UMAP plot illustrating the distribution of tumor‐infiltrating CD8^+^ T cells from the GSE200996 (n = 25) and GSE195832 (n = 8) datasets, color‐coded by the three broad cell groups (C1, C2, and C3). The plot shows a comparison of pretreatment and posttreatment samples, highlighting the expression levels of key markers, such as *GZMK*, *CXCL13*, *TIM3*, *CTLA4*, *LAYN*, and *MKI67*, in CD8^+^ T cells. B) Comparison of the proportions of three distinct clusters of CD8^+^ T cells between pretreatment and posttreatment samples. C) Schematic diagram illustrating the mouse subcutaneous transplantation tumor model of HNSCC treated with IgG as a control or aPD‐1 treatment on days 6, 8, 10, and 12 after tumor implantation (n = 4 per group). Created in https://BioRender.com. D) Tumor growth curves of the mice in different treatment groups; the arrows represent the time points of drug administration. E) Proportion of tumor‐infiltrating CD8^+^ T‐cell subsets in mice in different treatment groups. F) Flow cytometry analyzing the infiltration ratios of PD‐1^+^TCF‐1^−^CD8^+^ T (Tex^term^) cells, PD‐1^+^TCF‐1^+^CD8^+^ T (Tex^prog^) cells, and PD‐1^+^CD8^+^ T cells in response to IgG or aPD‐1 treatment. The data are presented as the mean ± SEM. ns, not significant, ^*^ *P* < 0.05, ^**^ *P* < 0.01, two‐tailed Student's t test. Pre, pretreatment; Post, posttreatment; Tex^term^ cells, terminally exhausted T cells; Tex^prog^ cells, precursor exhausted T cells; prolif, proliferation; aPD‐1, anti‐PD‐1; i.p., intraperitoneal injection; FCM, flow cytometry.

To assess the effects of aPD‐1 treatment on CD8^+^ T cell exhaustion in the TME of HNSCC, published scRNA‐seq datasets from GSE200996 and GSE195832 were utilized for bioinformatic analyses. The tumor‐infiltrating CD8^+^ T cells were obtained and classified into three broad cell groups^[^ ^18^ ^]^: the non‐exhausted cell subset (CD8‐C1‐non‐exhausted), the terminal exhausted cell subset (CD8‐C2‐Tex^term^), and the proliferative cell subset (CD8‐C3‐prolif) (**Figure** **1**A). Tex^term^ cells are characterized by high expression of exhaustion signature genes,^[^ ^19^ ^]^ including *PDCD1*, *CTLA4*, *CXCL13*, *HAVCR2*, and *LAYN*. Next, we compared the infiltration proportion of the CD8‐C2‐Tex^term^ subgroup in patients with HNSCC before and after treatment to further evaluate this concept. Our results showed that aPD‐1 treatment did not reduce the proportion of the CD8‐C2‐Tex^term^ subgroup; instead, it increased the proportion of cells in this subgroup (Figure 1B), supporting previous reports.^[^ ^20^, ^21^ ^]^

To further validate our findings, we generated a subcutaneous tumor model by inoculating SCC7 cells into C3HeN mice. The mice were treated with either an aPD‐1 or an isotype control IgG (Figure 1C). No statistically significant differences in tumor growth were observed between the aPD‐1 and IgG treatment groups (Figure 1D). To further characterize the tumor immune microenvironment, we performed flow cytometry to analyze CD8⁺ T cell subsets. APD‐1 treatment significantly reduced the proportion of Tex^prog^ cells and increased the frequency of CD8^+^PD‐1^−^ T cells. Conversely, there was no significant difference in the proportion of Tex^term^ cells between the aPD‐1 and IgG groups (Figure 1E,F).

These results collectively indicate that while aPD‐1 therapy may successfully reverse changes in the proportion of Tex^prog^ cells, it fails to reverse the proportion of Tex^term^ cells. This inability to reverse Tex^term^ cells may represent an underlying mechanism of immunotherapy resistance, highlighting the necessity of further investigation into the role of Tex^term^ cells in HNSCC.

### 2.2 Terminally Exhausted T Cells Contribute to the Progression of HNSCC

**Figure 2:**
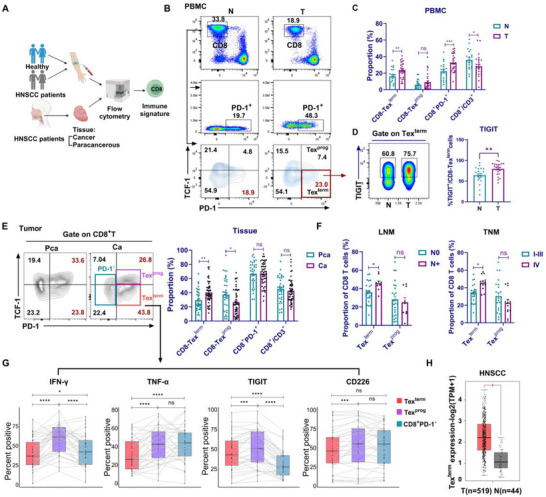
The infiltration level of Tex^term^ cells in tumor tissues, paracancerous tissues, and peripheral blood, and its relationship with tumor progression. A) Clinical sample collection and processing procedures. Figure created in https://BioRender.com. B) Flow cytometry gating strategy for the CD8^+^ T‐cell population. C) The proportions of CD8^+^ T cells and their subsets in the peripheral blood of HNSCC patients (n = 27) and healthy volunteers (n = 21) were statistically analyzed. D) The expression of the inhibitory receptor TIGIT on Tex^term^ cells was analyzed via flow cytometry. E) Flow cytometry showing the proportions of CD8^+^ T‐cell subsets in cancer (Ca) and paracancerous (Pca) tissues, and the color indicates the flow coil gates of different cell subsets. Statistical analysis of CD8^+^ T cells and their cell subsets in cancer tissue and paracancerous tissue (n = 34). F) The relationships between the proportions of Tex^term^ and Tex^prog^ in CD8^+^ T cells and lymph node metastasis (LNM), as well as TNM stage in patients, were statistically analyzed. G) Differences in the expression of IFN‐γ, TNF‐α, and the immune checkpoint receptors TIGIT and CD226 in each subset were statistically analyzed. H) The Tex^term^ cell expression signature in HNSCC tumor tissues (n = 519) compared with that in normal tissues (n = 44) from the TCGA database. The data are presented as the mean ± SEM; ns, not significant; ^*^ *P* < 0.05, ^**^ *P* < 0.01, ^***^ *P* < 0.001, ^****^ *P* < 0.0001. Significance was calculated by a two‐tailed Student's t‐test. HNSCC, head and neck squamous cell carcinoma; T, tumor; N, normal; TNM, tumor, node, metastasis; SEM, standard error of the mean.

To elucidate the significance of Tex^term^ cells in tumorigenesis and disease development, we prospectively collected tissues and peripheral blood from patients with HNSCC for flow cytometry analysis (**Figure** **2**A). The results showed a significant expansion of Tex^term^ (PD‐1^+^TCF‐1^−^CD8^+^ T) cells and PD‐1^+^CD8^+^ T cells, along with a concomitant reduction in total CD8⁺ T cells, in the peripheral blood of HNSCC patients compared to healthy volunteers. Notably, no significant difference was observed in the frequency of Tex^prog^ cells (PD‐1^+^TCF‐1^+^CD8^+^ T cells) (Figure 2B,C). Interestingly, the expression level of the immunosuppressive molecule TIGIT on Tex^term^ cells in the peripheral blood of HNSCC patients was higher than that in the peripheral blood of healthy volunteers (Figure 2D). Within the TME of HNSCC, elevated infiltration of Tex^term^ cells was detected in tumor tissues compared to paracarcinoma tissues, accompanied by a corresponding decrease in Tex^prog^ cells infiltration, a finding consistent with TCGA analysis (Figure 2E,H). Furthermore, the infiltration levels of Tex^term^ cells were significantly higher in patients with lymph node metastasis (LNM) and advanced clinical stage, highlighting their potential involvement in disease progression (Figure 2F). Additionally, Tex^term^ subsets produced significantly less IFN‐γ, TNF‐α, and CD226 compared to both Tex^prog^ and PD‐1^−^CD8⁺ T cell subsets (Figure 2G). In contrast, Tex^term^ subsets expressed higher levels of the immunosuppressive receptor TIGIT than PD‐1^−^CD8⁺ T cells (Figure 2G).

### 2.3 Single‐Cell RNA‐Seq Analysis Revealed the Role of Terminally Exhausted T Cells in Immunotherapy Resistance in HNSCC

**Figure 3:**
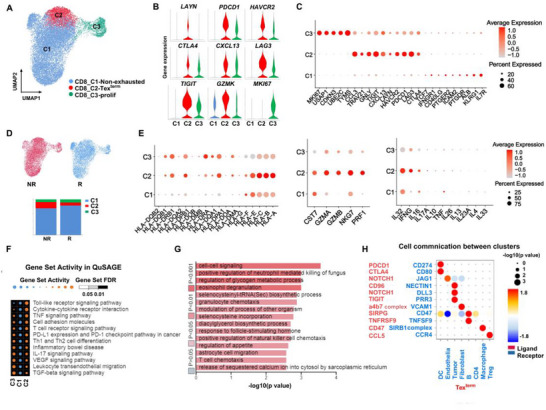
Single‐cell profiling of CD8^+^ T cells in the tumor microenvironment of HNSCC. A) UMAP plot of reclustered CD8^+^ T cells showing 3 clusters annotated in different colors from the GSE234933 (n = 14). B) Violin plots showing *LAYN*, *PDCD1, HAVCR2, CTLA4, CXCL13, LAG3, TIGIT, GZMK*, and *MKI67* expression in 3 clusters of CD8^+^ T cells. C) Dot plot illustrating the average expression levels and proportions of differentially expressed genes across three CD8^+^ T‐cell clusters. The size of each dot represents the percentage of cells expressing the gene, while the color indicates the average gene expression level per cell. D) UMAP plot and stacked bar chart displaying the proportions of three CD8^+^ T‐cell subpopulations in the immune therapy non‐responder (NR) group and the responder (R) group. E) Dot plot showing the expression of the genes encoding type II genes associated with the response, cytokine, and interleukin genes in different types of T cells. The dot size represents the percentage of cells expressing the gene, and the color represents the average per‐cell gene expression level. F) Gene set activity analysis via QuSAGE for three clusters of CD8^+^ T cells. G) Gene Ontology (GO) enrichment analysis of CD8‐C2‐Tex^term^ cells. H) Cell–cell interactions between Tex^term^ cells and other types of cells in the tumor microenvironment through ligand and receptor binding. Tex^term^ cells, terminally exhausted T cells; prolif, proliferation.

Considering the above findings, we sought to determine whether Tex^term^ cell infiltration contributes to immunotherapy resistance. We analyzed the GSE234933 scRNA‐seq dataset to compare the abundance of Tex^term^ cells between responders and non‐ responders to aPD‐1 therapy. Using expression levels of key marker genes, we performed clustering analysis on CD8⁺ T cells and classified them into three distinct subsets: CD8‐C1‐non‐exhausted T cells (characterized by high expression of *GZMK*, *IL7R*, *KLRG1*, *TCF7*, and *IFNGR1*), CD8‐C2‐Tex^term^ cells (enriched in inhibitory checkpoints including *LAYN*, *HAVCR2*, *PDCD1*, *CXCL13*, *LAG3*, *TIGIT*, and *CTLA4*), and CD8‐C3‐prolif cells (exhibiting elevated expression of *MKI67*, *USAP1*, and *CDKN3*) (**Figure** **3**A–C; Table S1, Supporting Information). As hypothesized, CD8‐C2‐Tex^term^ cells were more abundant in nonresponders than in responders (Figure 3D; Table S2, Supporting Information). Functional profiling further revealed elevated expression of multiple HLA class genes (including *HLA‐DQB1*, *HLA‐DRB1*, *HLA‐DPB1*, *HLA‐DQA1*, *HLA‐F*, *HLA‐E*, *HLA‐C*, *HLA‐B*, and *HLA‐A*) in this subset, suggesting a potential role in antigen presentation and immune regulation. Additionally, CD8‐C2‐Tex^term^ cells exhibited high expression of cytotoxic molecules (*CST7*, *GZMA*, *PRF1*, and *NKG7*) and cytokines (*IL32*, *IL16*, *IFNG*, and *IL7A*), but reduced expression of *TNF‐α* (Figure 3E).

To delve deeper, we employed gene set activity analysis using QuSAGE, which revealed that the CD8‐C2‐Tex^term^ cell subsets were predominantly enriched in signaling pathways, including the Toll‐like receptor pathway, cytokine‒cytokine receptor interaction, PD‐L1 expression and the PD‐1 checkpoint pathway in cancers (Figure 3F). Moreover, further GO analysis revealed that most immune‐related pathways, including cell‒cell signaling, positive regulation of neutrophil‐mediated killing of fungus, eosinophil degranulation, granulocyte chemotaxis, positive regulation of natural killer cell chemotaxis, and T‐cell chemotaxis, were enriched in CD8‐C2‐Tex^term^ cells (Figure 3G; Table S3, Supporting Information). Finally, we used CellPhoneDB to investigate cell‒cell communication between Tex^term^ cells and other cell types in tumors. The results showed that Tex^term^ cells might trigger the recruitment of regulatory T cells (Tregs) into the TME via CCL5‐CCR4 binding, thus promoting immune suppression, which is consistent with the literature.^[^ ^22^ ^]^ Additionally, Tex^term^ cells might facilitate tumor cell immune evasion through the TIGIT–PRR3 signaling axis (Figure 3H).

These findings demonstrate the important role of Tex^term^ cells in immunotherapy resistance and tumor immune escape in HNSCC.

### 2.4 High Infiltration of Terminally Exhausted T Cells Results in a Significant Immunotherapy Resistance Phenotype

We next performed a retrospective clinical study including 40 patients who received aPD‐1 therapy at the First Affiliated Hospital of Sun Yat‐sen University between December 2020 and June 2023. According to RECIST 1.1 criteria, patients were categorized into a responder group (PR + CR, n = 26) and a non‐responder group (PD + SD, n = 14). As shown in **Table** **1**, patients in the non‐responder group were strongly correlated with treatment options, age, and the proportion of PD‐1^+^TCF‐1^−^CD8^+^ T (Tex^term^) cells in both the stromal and tumor areas.

**Table 1:** Baseline characteristics of 40 HNSCC patients receiving anti‐PD‐1 therapy.

Characteristics	N(%)/Median (Interquartile Range)			*P* value
	Responder (PR + CR, n = 26)	Non‐responder (SD + PD, n = 14)	Total (n = 40)	
Median OS (months)	25.08 (13.65, 31.40)	9.93 (4.48, 16.79)	17.77 (9.92, 29.93)	0.01
Sex				
Female	0 (0.00)	1 (7.14)	1 (2.50)	0.35
Male	26 (100.00)	13 (92.86)	39 (97.50)	
Age (year)				0.00
<62	14 (53.85)	5 (35.71)	19 (47.50)	
≥62	12(46.15)	9 (64.294)	21 (52.50)	
Drinking				0.97
Yes	15 (57.69)	8 (57.14)	23 (57.50)	
No	11 (42.31)	6 42.86)	17 (42.50)	
Smoking				0.97
Yes	17 (65.38)	10 (71.43)	27 (67.50)	
No	9 (34.62)	4 (28.57)	13 (32.50)	
Tumor location				0.39
Larynx	1 (3.85)	3 (21.43)	4 (10.00)	
Hypopharynx	14 (53.85)	6 (42.86)	20 (50.00)	
Oropharynx or others	6 (23.08)	3 (21.43)	9 (22.50)	
More than one site	5 (19.23)	2 (14.29)	7 (17.50)	
R/M				0.78
No	21 (80.77)	10 (71.43)	31 (77.50)	
Yes	5 (19.23)	4 (28.57)	9 (22.50)	
Treatment				0.03
TP+K	24 (92.31)	8 (60.00)	32 (82.14)	
K	2 (5.56)	6 (40.00)	8 (17.86)	
CPS				0.26
0	3 (11.54)	4 (28.57)	7 (17.50)	
1‐19	16 (61.54)	8 (57.14)	24 (60.00)	
≥20	7 (26.92)	2 (14.29)	9 (22.50)	
P16 status				0.98
Positive	7 (19.44)	3 (21.42)	10 (25.00)	
Negative	15 (41.67)	6 (42.86)	21 (52.50)	
Unknown	4 (11.11)	5 (35.71)	9 (22.50)	
Stroma PD‐1^+^TCF‐1^−^/CD8^+^ (%)	17.00 ± 10.70	35.00 ± 13.90	23.00 ± 14.40	0.00
Tumor PD‐1^+^TCF‐1^−^/CD8^+^ (%)	22.00 ± 15.10	34.00 ± 14.60	26.00 ± 15.80	0.03
TME PD‐1^+^TCF‐1^−^/CD8^+^ (%)	19.00 ± 11.40	29.00 ± 13.40	23.00 ± 12.80	0.03
Stroma PD‐1^+^TCF‐1^+^/CD8^+^ (%)	18.21 ± 13.16	22.08 ± 10.31	19.57±12.24	0.35
Tumor PD‐1^+^TCF‐1^+^/CD8^+^ (%)	16.00 ± 14.00	19.00 ± 12.00	17.00 ± 13.30	0.50
TME PD‐1^+^TCF‐1^+^/CD8^+^ (%)	17.00 ± 11.82	20.12 ± 10.31	18.09 ± 11.08	0.40
Survival state				0.00
Alive	22 (84.62)	10 (71.43)	32 (80.00)	
Dead	4 (15.38)	4 (28.57)	8 (20.00)	

Abbreviations: OS, overall survival; PR, partial response; CR, complete response; PD, progressive disease; SD, stable disease; R/M, recurrent or metastatic; CPS, combined positive score; TP, taxol + cisplatin; K, keytruda.

**Figure 4:**
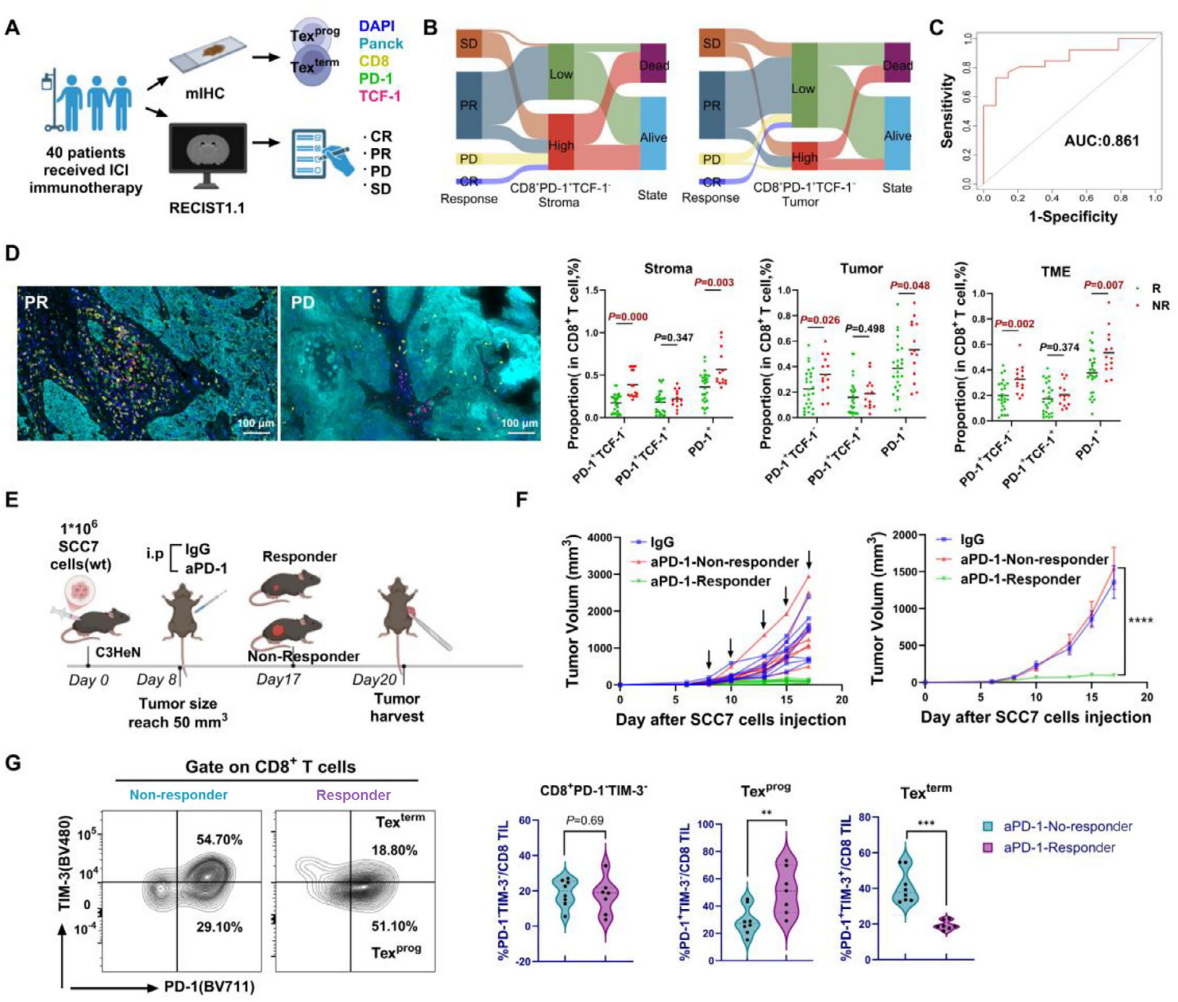
TME infiltration of CD8^+^ T‐cell subsets in the aPD‐1 treatment responder group and non‐responder group was assessed. A) Summary of the procedure used to evaluate CD8^+^ T‐cell subset infiltration in the TME of 40 patients receiving immune checkpoint inhibitor (ICI) treatment, and the RECIST 1.1 criteria for assessing immunotherapy efficacy. Created in https://BioRender.com. B) Sankey diagram illustrating patient transfer during immunotherapy, infiltration of PD‐1^+^TCF‐1^−^CD8^+^ T (Tex^term^) cells within the stroma or tumor, and survival status in the clinical cohort. C) ROC analysis of the ability of Tex^term^ cell infiltration levels to predict the efficacy of immunotherapy. D) Multiplex immunofluorescence staining of tumor tissues from HNSCC patients was performed to detect CD8 (yellow), PD‐1 (green), TCF‐1 (red), Pan‐CK (light green), and DAPI (blue) expression, with a scale bar of 100 µm. The infiltration of different subsets of CD8^+^ T cells in the stroma, tumor, and TME between the responder (CR+PR) groups (n = 26) and the non‐responder (SD+PD) groups (n = 14) was statistically analyzed. E) Flow chart of the in vivo animal experiments. Created in https://BioRender.com. F) Tumor volume curves of SCC7 tumor‐bearing C3HeN mice treated with IgG as a control (n = 7) or aPD‐1 (n = 15) twice a week (arrows represent time points of administration). The mice in the aPD‐1 treatment group were divided into a responder group and a non‐responder group according to tumor size. Significance was calculated by two‐way ANOVA. G) The proportions of CD8^+^ T‐cell subsets in the tumor microenvironment of mice in the aPD‐1 treatment responder group and non‐responder group were analyzed by flow cytometry. The data are presented as the mean ± SEM. ^**^ *P* < P0.01, ^***^ *P* < 0.001; two‐tailed Student's t test. PR, partial response; CR, complete response; PD, progressive disease; SD, stable disease; Tex^term^ cells, terminally exhausted T cells; Tex^prog^ cells, precursor exhausted T cells; AUC, area under the curve; TME, tumor microenvironment; R, responder; NR, non‐responder; i.p., intraperitoneal injection; wt, wild type; aPD‐1, anti‐PD‐1.

Pretreatment biopsy samples were analyzed using mIHC to assess the proportions of CD8^+^ T cell subsets in the TME of HNSCC patients (**Figure** **4**A; Table S4, Supporting Information). Specifically, our results revealed that patients with lower levels of Tex^term^ cells were more likely to respond positively to the treatment (Figure 4B). Most importantly, the infiltration level of Tex^term^ cells could serve as a predictive marker for the immunotherapy response, demonstrating high sensitivity (AUC = 0.861; Figure 4C). Furthermore, mIHC analysis revealed a notable increase in Tex^term^ cells within both the stromal area and tumor area in the non‐responder group compared with the responder group (stromal area: 35.00% ± 13.90 vs 17.00% ± 10.70, *P* < 0.01; tumor area: 34.00% ± 14.60 vs 22.00% ± 15.10, *P* = 0.026; Figure 4D and Table 1). This trend also extended to PD‐1^+^CD8^+^ T cells, which displayed similar elevation patterns. Conversely, the number of Tex^prog^ cells was not significantly different between the responder group and non‐responder group (*P* > 0.05, Figure 4D and Table 1).

To further validate our findings, we conducted mouse xenograft model experiments in which both aPD‐1 and IgG (as an isotype control) were administered (Figure 4E). Mice in the aPD‐1 group with tumor growth curves demonstrating a proliferation rate lower than that of the IgG group were classified as responders to immunotherapy, whereas those with a higher proliferation rate were categorized as non‐responders. (Figure 4F). Using flow cytometry, we analyzed the proportions of cell subsets of CD8^+^ T cells present in the TME of each group. Our findings revealed a significant increase in the infiltration of Tex^term^ cells and a decrease in Tex^prog^ cells in the immunotherapy non‐responder cohort compared with the responder cohort (*P* < 0.01, Figure 4G).

These results suggest that Tex^term^ cells may be pivotal factors contributing to immune therapy resistance in HNSCC. Thus, suppressing the differentiation of CD8 effector T cells into Tex^term^ cells may represent an effective therapeutic strategy for enhancing the immune response against tumors.

### 2.5 The Proportion of Terminally Exhausted T Cells is an Independent Prognostic Factor for Patients Receiving aPD‐1 Therapy

**Figure 5:**
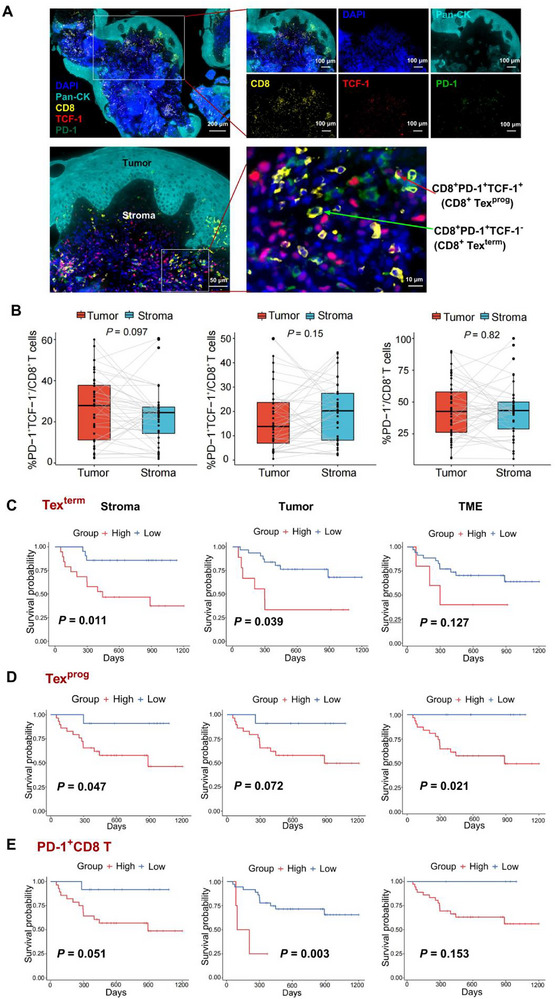
The infiltration of CD8^+^ T‐cell subsets in the tumor stroma, tumor nests, and TME and their correlation with the prognosis of HNSCC. A) Multiplex immunofluorescence images showing the markers for CD8 (yellow), PD‐1 (green), TCF‐1 (red), Pan‐CK (light green), and DAPI (blue). Scale bar, 200 µm. The framed areas are shown below at a higher magnification. The area with positive Pan‐CK expression was the tumor nest, and the rest was considered the stroma; red arrows indicate PD‐1^+^TCF‐1^+^CD8^+^ T (Tex^prog^) cells; green arrows indicate PD‐1^+^TCF‐1^−^CD8^+^ T (Tex^term^) cells. B) The infiltration of CD8^+^ T‐cell subsets in the tumor area and stroma area of HNSCC patients was compared separately via a two‐tailed Student's paired t‐test. C–E) Survival curves showing the associations between the infiltration level of CD8^+^ T‐cell subsets in the stroma area, tumor area, and TME and overall survival in HNSCC patients in the retrospective clinical cohort (log‐rank test). Survival curves for Tex^term^ (upper panel), Tex^prog^ (middle panel), and PD‐1^+^ CD8 (lower panel) are shown, along with the corresponding *P* values. The high‐ and low‐expression groups were divided based on the optimal cutoff value determined by the ggsurvplot R package. HNSCC, head and neck squamous cell carcinoma; Tex^term^ cells, terminally exhausted T cells; Tex^prog^ cells, precursor exhausted T cells; TME, tumor microenvironment.

MIHC analysis revealed significantly greater infiltration of CD8^+^ T cells in the stromal area than in the tumor area. However, when the proportions of CD8^+^ T‐cell subsets were analyzed, no significant differences were found in the proportions of Tex^term^, Tex^prog^ and PD‐1^−^CD8^+^ T‐cell subsets in either the tumor or stromal areas (**Figure** **5**A,B). Utilizing the log‐rank method, we performed survival analyses of CD8^+^ T‐cell subsets in the stromal area, tumor area, as well as the entire TME. Our findings indicated that lower infiltration levels of Tex^term^ and Tex^prog^ cells in the stromal area, along with reduced Tex^term^ and PD‐1⁺ CD8⁺ T cell infiltration in the tumor area, were significantly associated with improved prognosis in HNSCC (Figure 5C–E). Specifically, reduced stromal or tumoral Tex^term^ infiltration, but not in the whole TME, correlated with longer overall survival (OS). Furthermore, patients exhibiting low infiltration of Tex^term^ and Tex^prog^ cells in the stroma, low Tex^prog^ in the TME, or low PD‐1^+^CD8^+^ T cells in the stromalp or tumor area tended to have better progression‐free survival (PFS) (Figure S1, Supporting Information).

We subsequently performed univariate analyses and revealed that patients with recurrence/metastasis (R/M), those receiving immunotherapy alone, and those with low infiltration levels of Tex^term^ cells in both the stromal and tumor areas exhibited better outcomes (**Table** **2**). Multivariate Cox regression analysis revealed that R/M and Tex^term^ cell infiltration levels within the stromal area were independent prognostic factors in patients (Table 2).

**Table 2:** Univariate and multivariate Cox regression analyses of factors influencing OS.

	Univariable		Multivariate	
	HR (95% CI)	*P*‐value	HR (95% CI)	*P*‐value
Age(≥62 vs <62)	1.931 (0.646‐5.769)	0.239		
Drinking (Yes vs No)	1.325 (0.441‐3.978)	0.616		
Smoking (Yes vs No)	1.823 (0.507‐6.551)	0.358		
R/M(Yes vs No)	3.544 (1.211‐10.372)	0.021	5.413(1.705‐17.178)	0.004
Treatment (TP+K vs K)	0.234 (0.080‐0.679)	0.008		
CPS		0.327		
1‐19 vs 0	0.183 (0.017‐2.034)	0.167		
≥20 vs 0	0.296 (0.027‐3.285)	0.321		
P16 status		0.975		
Positive vs Negative	0.891 (0.079‐10.074)	0.926		
unknown vs Negative	1.185 (0.061‐22.918)	0.911		
Stroma‐PD‐1^+^TCF‐1^−^/CD8^+^(%, high vs low)	4.543 (1.266‐16.302)	0.020	5.028 (1.348‐18.753)	0.016
Tumor‐PD‐1^+^TCF‐1^−^/CD8^+^(%, high vs low)	2.920(1.005‐8.485)	0.049	3.071 (0.986‐9.568)	0.053
TME‐PD‐1^+^TCF‐1^−^/CD8^+^(%, high vs low)	2.397 (0.666‐8.622)	0.181		
Stroma‐PD‐1^+^TCF‐1^+^/CD8^+^(%, high vs low)	6.245 (0.804‐48.502)	0.080		
Tumor‐PD‐1^+^TCF‐1^+^/CD8^+^(%, high vs low)	5.304 (0.692‐40.669)	0.108		
TME‐PD‐1^+^TCF‐1^+^/CD8^+^(%, high vs low)	34.038 (0.232, 4985.371)	0.166		

Abbreviations: OS, overal survival; HR, hazard ratio; CI, confidence interval; R/M, recurrent or metastatic; TP, taxol and cisplatin; K, keytruda; CPS, combined positive score; TME, tumor microenvironment.

Subsequently, to evaluate the prognostic value of stromal Tex^term^ cells across different patients with combined positive score (CPS categories and p16 status, we performed subgroup analyses. The results revealed that among patients with CPS scores of 1–19 and those with p16‐positive status, low infiltration of stromal Tex^term^ cells was still associated with significantly better prognosis (*P* < 0.05). Moreover, a consistent trend was observed in subgroups with CPS = 0, CPS ≥ 20, and p16‐negative disease, although these differences did not reach statistical significance, likely due to the limited sample size within these specific cohorts (Figure S2, Supporting Information).

These results collectively suggest that high infiltration of Tex^term^ cells may promote HNSCC progression and highlight the potential value of Tex^term^ cells infiltration as an indicator of patients’ clinical outcomes.

### 2.6 The Infiltration Level of Terminally Exhausted T Cells is Closely Correlated with PD‐L1 Expression on Tumor Cells

**Figure 6:**
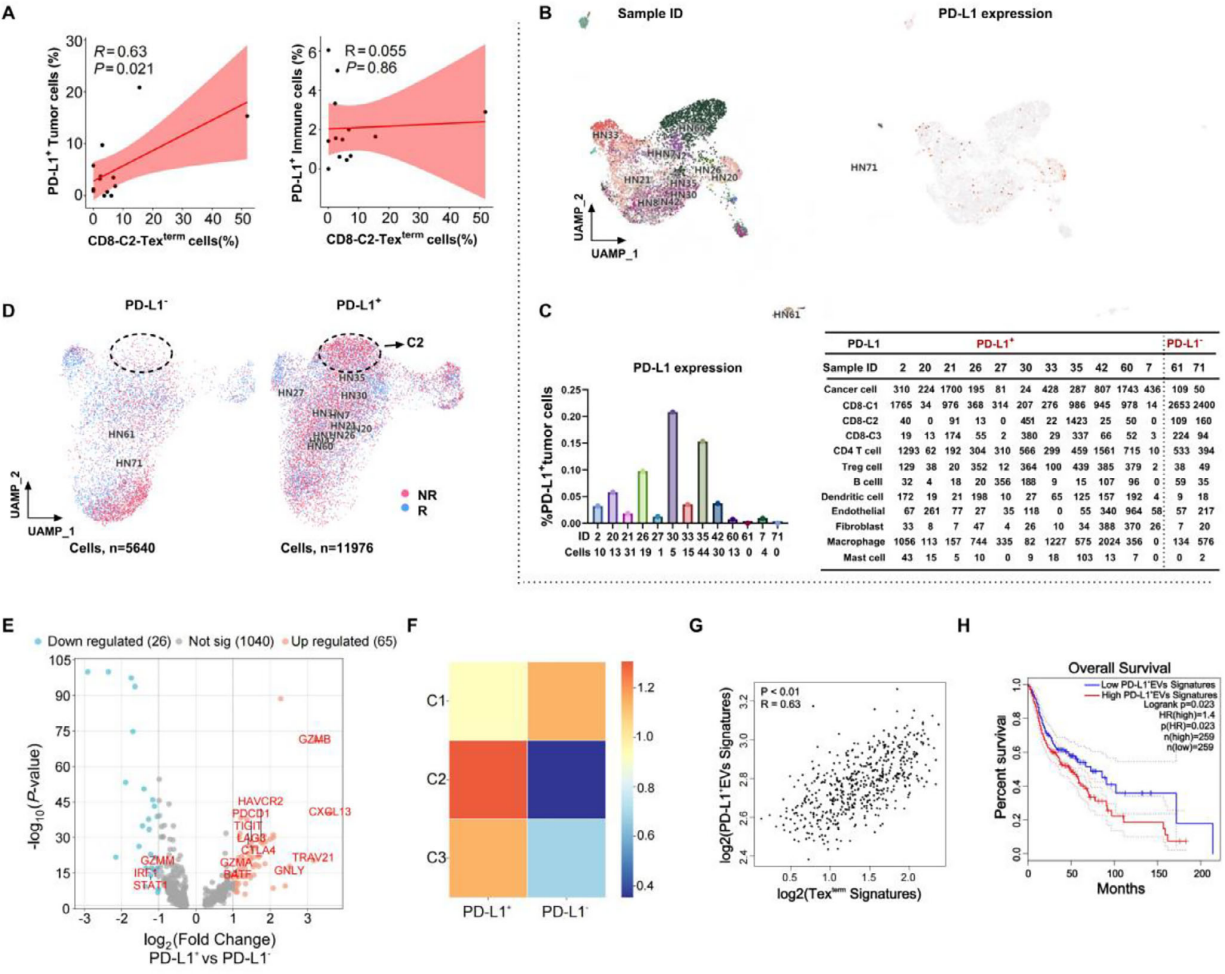
Complex regulatory networks between PD‐L1 expression on tumor cells and the infiltration level of Tex^term^ cells in the HNSCC TME from the GSE234933 single‐cell RNA sequencing cohort. A) Correlation of the CD8‐C2‐Tex^term^ cell subpopulation infiltration ratio with PD‐L1^+^ tumor cells or PD‐L1^+^ immune cells. B) Cancer cell clusters of HNSCC based on sample IDs and PD‐L1 expression on individual cancer cells from different HNSCC samples (red dots represent cells positive for PD‐L1). C) Bar graph of PD‐L1^+^ tumor cells in different tumor samples (left panel). Single‐cell compositions and cell numbers for each sample ID (n = 13) with either PD‐L1^+^ tumor group (samples 2, 20, 21, 26, 27, 30, 33, 35, 42, 60, and 7) or PD‐L1^−^ tumor group (samples 61 and 71). D) UMAP of CD8^+^ T cells in PD‐L1^+^ tumor group or PD‐L1^−^ tumor group colored according to treatment response conditions (the dashed line indicates the location of the CD8‐C2‐Tex^term^ subset). E) Differentially expressed genes of CD8‐C2‐Tex^term^ subset in PD‐L1^+^ tumor group compared with PD‐L1^−^ tumor group. F) Heatmaps of CD8^+^ T‐cell subsets (C1, C2 and C3) in PD‐L1^+^ tumors and PD‐L1^−^ tumors. G) Correlation analysis of the Tex^term^ signature and PD‐L1^+^ EV signature using data from the TCGA database. H) Survival differences between the high‐PD‐L1^+^ EVs group and the low‐PD‐L1^+^ EVs group in the TCGA‐HNSCC cohort (log‐rank test). Tex^term^ cells, terminally exhausted exhaustion of T cells; TCGA, The Cancer Genome Atlas; HNSCC, head and neck squamous cell carcinoma; EVs, extracellular vesicles.

Recent studies have shown that PD‐L1 expression on both cancer cells and non‐cancer cells significantly influences patient outcomes.^[^ ^23^, ^24^, ^25^ ^]^ Specifically, PD‐L1 signaling on CD8^+^ T cells can induce a nonreactive T‐bet‐IFN‐γ‐phenotype.^[^ ^23^ ^]^ Therefore, we analyzed the relationship between Tex^term^ cells and PD‐L1 expression on tumor cells vs immune cells using scRNA‐seq data. Intriguingly, our results revealed a significant positive association between PD‐L1 expression on tumor cells and the density of infiltrating Tex^term^ cells (R = 0.63, *P* = 0.021, **Figure** **6**A). To further validate this, we stratified patients into PD‐L1^+^ tumor group (samples 2, 20, 21, 26, 17, 30, 33, 35, 42, 60, and 7) and PD‐L1^−^ tumor group (samples 61 and 71) based on the PD‐L1 expression on tumor cells (Figure 6B,C; Table S5, Supporting Information). The proportions of various types of tumor‐infiltrating cells were assessed in the PD‐1^+^ tumor group and the PD‐1^−^ tumor group (Figure 6C; Table S6, Supporting Information). Notably, compared with the PD‐L1^−^ tumor group, the upregulated differentially expressed genes (DEGs) in the CD8‐C2‐Tex^term^ subset from PD‐L1⁺ tumor group included exhaustion‐related genes such as *HAVCR2, CXCL13, PDCD1, TIGIT, LAG3, BATF, GNLY*, and *CTLA4* (Figure 6E; Table S7, Supporting Information). Furthermore, greater infiltration of CD8‐C2‐Tex^term^ cells was observed in the PD‐L1^+^ tumor group than in the PD‐L1^−^ tumor group (Figure 6D,F). Our previous study revealed that tumor‐derived EVs carry more PD‐L1 than tumor cells, promoting the formation of an immunosuppressive microenvironment and possibly contributing to immunotherapy resistance.^[^ ^26^ ^]^ Further analysis of the TCGA database revealed a significant positive correlation between the signature of Tex^term^ cells and PD‐L1^+^ EVs (R = 0.63, *P* < 0.01, Figure 6G). Importantly, lower levels of PD‐L1^+^ EVs were associated with a better prognosis for HNSCC (Figure 6H).

### 2.7 PD‐L1 on Tumor‐Derived Extracellular Vesicles Drives Terminal Exhaustion of CD8+ T Cells by Upregulating BATF

**Figure 7:**
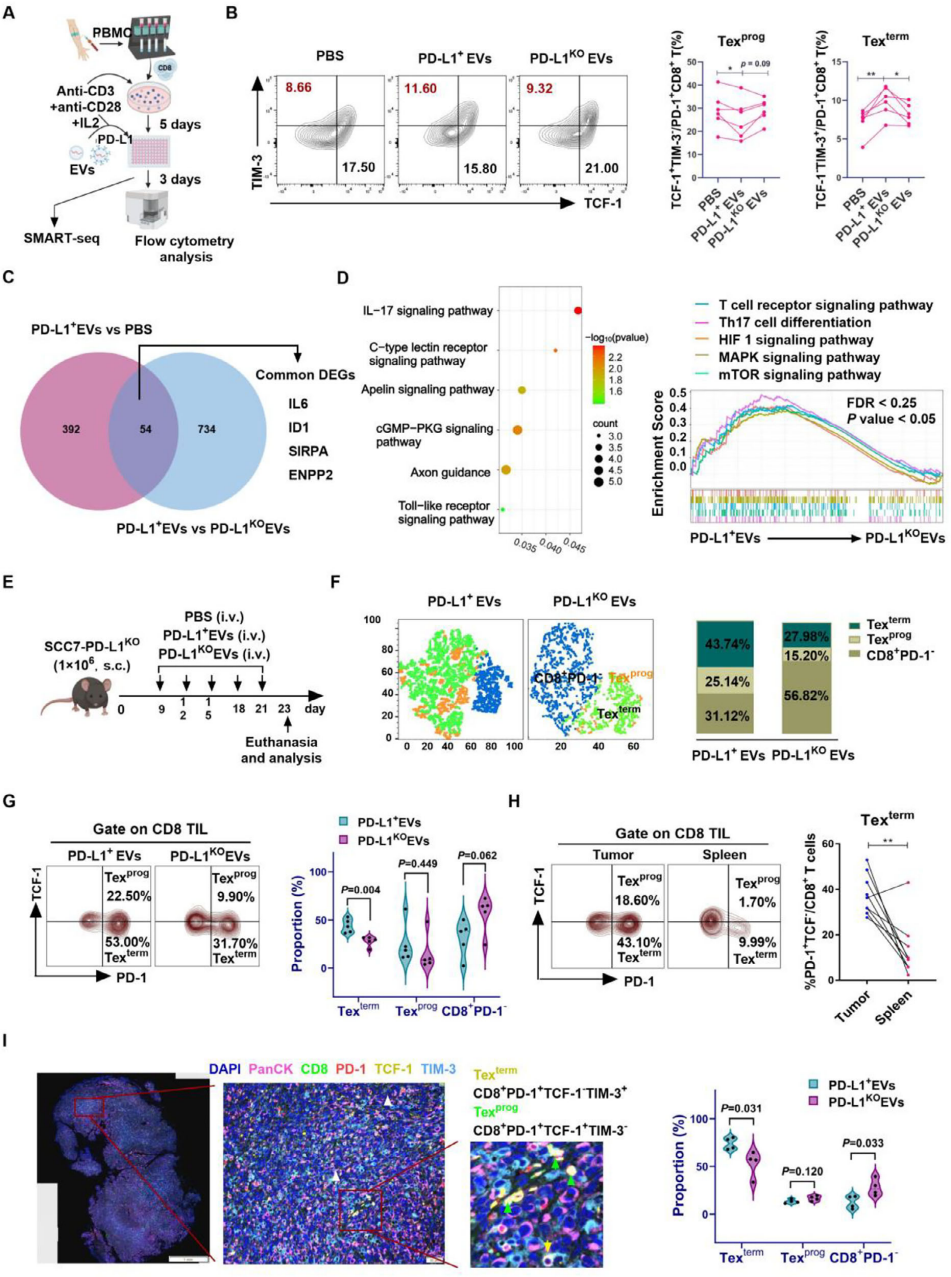
Effect of tumor‐derived PD‐L1^+^ EVs on the induction of Tex^term^ in the TME. A) Study design and technical flowchart of in vitro experiments. Created in https://BioRender.com. B) The effects of PBS, PD‐L1^+^ EVs, and PD‐L1^KO^ EVs treatment on the induction of CD8^+^ T‐cell subsets were assessed through flow cytometry analysis. The proportions of Tex^term^ and Tex^prog^ cell subsets across the different treatment groups were statistically analyzed. C) The intersection of differentially expressed genes (DEGs) between the two groups according to SMRT‐seq analysis. D) KEGG pathway analysis revealed the enrichment of several important pathways among the DEGs of PD‐L1^+^ EVs and PD‐L1^KO^ EVs, as did GSEA. E) Schematic diagram of the subcutaneously implanted tumor model, illustrating tail vein injection with PBS, PD‐L1^+^ EVs, or PD‐L1^KO^ EVs. (n = 5, each group). F) Density ViSNE and stacked plots of tumor‐infiltrating CD8^+^ T cells are presented, overlaid with the expression of three distinct cell subsets as determined by flow cytometry. G) Flow cytometry analysis of infiltrating CD8^+^ T‐cell subsets in the tumor microenvironment of mice that were administered PD‐L1^+^ EVs or PD‐L1^KO^ EVs via tail vein injection. H) Comparative analysis of infiltrating CD8^+^ T‐cell subsets in the tumor microenvironment and peripheral spleen of mice. I) Multiple immunohistochemistry results for pan‐CK (pink), CD8 (green), PD‐1 (red), TCF‐1 (yellow), TIM‐3 (blue), and DAPI in the TME of mice administered PD‐L1^+^ EVs and PD‐L1^KO^ EVs. Scale bar, 50 µm. The framed areas are shown below at a higher magnification; the yellow arrow indicates CD8^+^PD‐1^+^TCF‐1^−^TIM‐3^+^ T (Tex^term^) cells; the green arrows indicate CD8^+^PD‐1^+^TCF‐1^+^TIM‐3^−^ T (Tex^prog^) cells. Statistical analysis was performed for the tumor‐infiltrating CD8^+^ T‐cell subsets among the various groups of mice. Data are presented as the mean ± SEM, ^*^ *P* < 0.05, ^**^ *P* < 0.01; significance was calculated by two‐tailed Student's t test or paired t test. PBMC, peripheral blood mononuclear cell; EVs, extracellular vesicles; PBS, phosphate‐buffered saline; TIL, tumor‐infiltrating lymphocyte; Tex^term^ cells, terminally exhausted exhaustion of T cells; Tex^prog^ cells, precursor exhausted T cells.

To further validate the effects of tumor‐derived PD‐L1^+^ EVs on Tex^term^ cells generation, CD8⁺ T cells from healthy volunteers were co‐cultured with PBS, PD‐L1⁺ EVs, or PD‐L1^KO^ EVs in vitro (**Figure** **7**A). Compared with the PD‐L1^+^ EVs group, the PD‐L1^KO^ EVs group showed a significant reduction in Tex^term^ cell production and an increase in Tex^prog^ cells (Figure 7B). CD8^+^ T cells from the PBS, PD‐L1⁺ EVs, and PD‐L1^KO^ EVs treatment groups were subsequently subjected to SMART‐seq analysis. Results indicated that DEGs associated with inter‐group comparisons, such as *IL6, ID1, SIRPA*, and *ENPP2*, were highly correlated with established mechanisms of T‐cell exhaustion (Figure 7C; Tables S8 and S9, Supporting Information). KEGG and GSEA enrichment analysis of DEGs between PD‐L1⁺ EVs group and PD‐L1^KO^ EVs group revealed the enrichment in the PD‐L1⁺ EVs treatment group for pathways including: IL‐17 signaling, C‐type lectin receptor signaling, Toll‐like receptor signaling, cGMP‐PKG signaling, T‐cell receptor signaling, Th17 cell differentiation, HIF‐1 signaling, MAPK signaling, and metabolic reprogramming pathways, such as Apelin signaling and mTOR signaling (Figure 7D; Table S10, Supporting Information). These findings suggest that PD‐L1^+^ EVs promote T cell exhaustion by activating inflammatory signaling, suppressing stem‐like properties, and inducing metabolic reprogramming.

Furthermore, in animal experiments, subcutaneous tumor models were established using PD‐L1^KO^ SCC7 cells, followed by tail vein administration of PBS, PD‐L1^+^ EVs, or PD‐L1^KO^ EVs (Figure 7E). Tumor growth curves were evaluated as previously described.^[^ ^26^ ^]^ Flow cytometric and mIHC analyses of CD8^+^ T cell subsets in the TME confirmed a significantly lower proportion of Tex^term^ cells in PD‐L1^KO^ EV‐treated mice compared to PD‐L1⁺ EV‐treated mice (Figure 7F,G,I). while no significant difference was observed in Tex^prog^ cell proportions (Figure 7F,G,I). Additionally, Tex^term^ cell infiltration in the TME was significantly greater than in the spleen, comprising the majority of the CD8⁺ T cell population (Figure 7H). These findings suggest that PD‐L1⁺ EVs effectively mediate the T cell exhaustion process.

**Figure 8:**
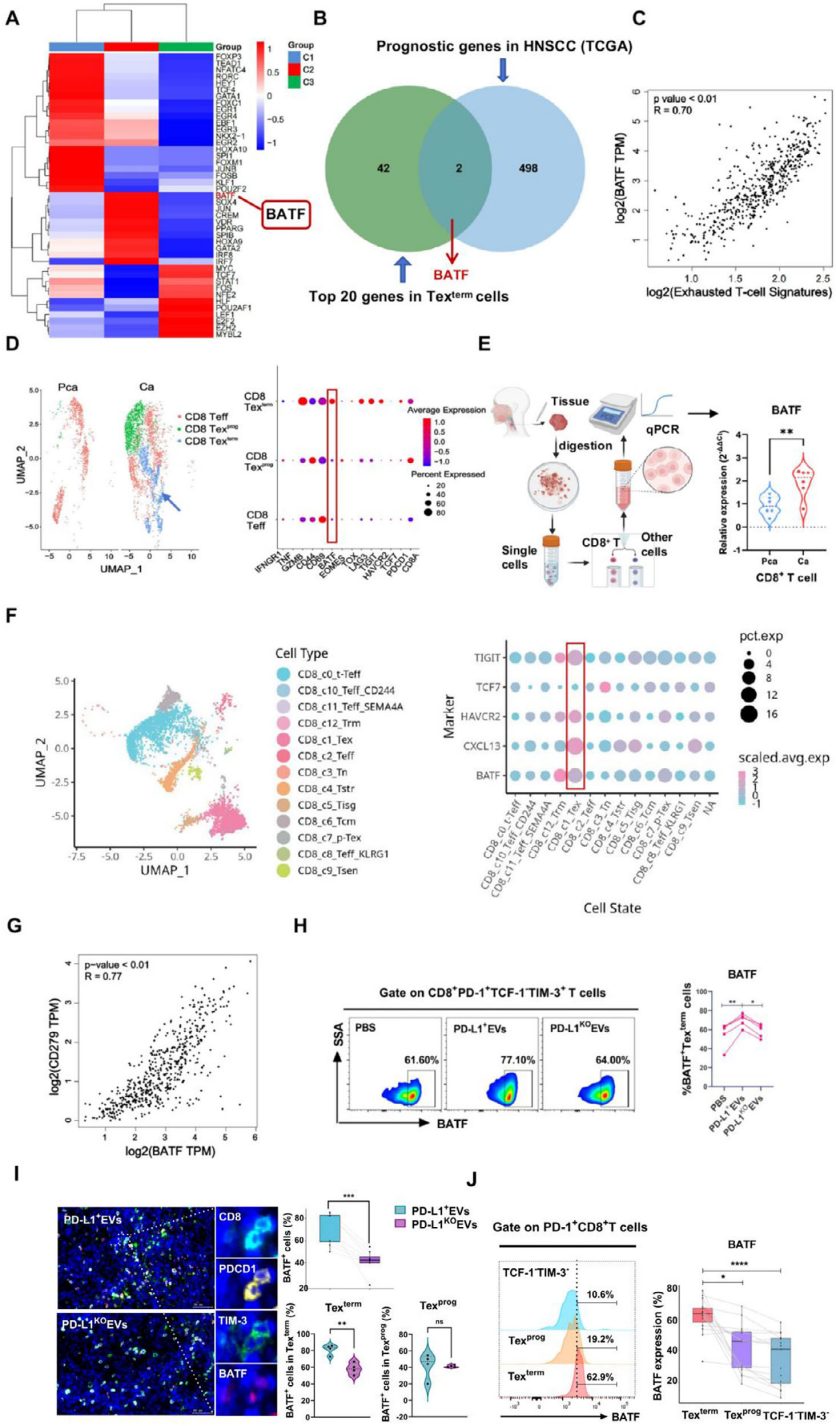
Tumor‐derived PD‐L1^+^ EVs may promote the terminal exhaustion of CD8^+^ T cells by up‐regulating the expression of transcription factor BATF. A) Top 20 enriched transcription factors for CD8^+^ T cell subsets. B) Venn diagram of the intersection of the top 20 enriched transcription factors of the Tex^term^ cell subsets and the patient's prognostic related genes of HNSCC in TCGA. C) Correlation between the signature of Tex^term^ cells and the expression of BATF using the TCGA database. D) UMAP and bubble plot of gene expression in CD8^+^ T cell subsets were analyzed using scRNA‐seq from six primary tumor tissues and two adjacent normal tissues of HNSCC. E) Expression level of the BATF gene in CD8^+^ T cells in tumor tissues and adjacent normal tissues was analyzed by qPCR (n = 6). F) UMAP and bubble plot of gene expression in CD8^+^ T cell subsets were anlyzed (https://singlecell.mdanderson.org/TCM/embedding). G) Correlation between the expression of *BATF* mRNA and *CD279* mRNA using the TCGA database. H) BATF expression in CD8 Tex^term^ cells induced by PBS, PD‐L1^+^ EVs, and PD‐L1^KO^ EVs was compared. I) Multiple immunohistochemistry results for CD8 (blue), PD‐1 (yellow), TIM‐3 (green), BATF (red), and DAPI in the TME of mice administered PD‐L1^+^ EVs and PD‐L1^KO^ EVs (n = 4). J) BATF expression in CD8^+^ T cell subsets after treatment with PD‐L1^+^ EVs or PD‐L1^KO^ EVs in vitro experiments, as determined by flow cytometry. Data are presented as the mean ± SEM, ^*^ *P* < 0.05, ^**^ *P* < 0.01; significance was calculated by two‐tailed paired or unpaired Student's t test, or One‐Way ANOVA analysis. EVs, extracellular vesicles; PBS, phosphate‐buffered saline; Tex^term^ cells, terminally exhausted exhaustion of T cells; Tex^prog^ cells, precursor exhausted T cells.

To elucidate the underlying mechanism, we intersected the top 20 transcription factors enriched in Tex^term^ cells with prognosis‐associated genes in HNSCC patients (Table S11, Supporting Information). This identified BATF as a key transcription factor enriched in the CD8‐C2‐Tex^term^ subset (**Figure** **8**A), which was strongly associated with patient prognosis (Figure 8B). TCGA database analysis confirmed a significant positive correlation between *BATF* mRNA expression and Tex^term^ cell signature scores (R = 0.70, *P* < 0.01; Figure 8C). ScRNA‐seq recluster analysis of CD8^+^ T cells from 6 HNSCC tumor tissues and 2 adjacent tissues revealed higher infiltration of Tex^term^ cells and significantly elevated BATF expression in CD8‐Tex^term^ subsets within tumors (Figure 8D). This was validated by qPCR showing higher BATF expression in CD8+ T cells derived from the tumor tissuethan that in the normal tissue (Figure 8E). Analysis of a public HNSCC scRNA‐seq dataset^[^ ^27^ ^]^ confirmed BATF enrichment specifically in CD8‐Tex^term^ cells (Figure 8F). To investigate PD‐L1^+^ EVs regulation of BATF, a strong correlation was observed between *BATF* and *CD279* (PD‐1) mRNA expression (R = 0.77, *P* < 0.01, Figure 8G). Flow cytometry confirmed that PD‐L1⁺ EVs significantly upregulated BATF expression in CD8‐Tex^term^ cells compared to PBS‐treated controls and PD‐L1^KO^ EVs (Figure 8H). Furthermore, in the subcutaneous tumor model, PD‐L1^+^ EVs treatment led to a significant increase in BATF^+^ Tex^term^ cells compared to PD‐L1^KO^ EVs treatment, while no such difference was observed in the Tex^prog^ subset (Figure 8I). Consistent with this, both in vivo and in vitro CD8^+^ T cell exhaustion models showed consistently higher BATF expression in Tex^term^ cells than in Tex^prog^ cells, demonstrating its specific enrichment in the terminally exhausted subset (Figure 8I,J).

## 3 Discussion

Head and neck squamous cell carcinoma (HNSCC) remains a profound global oncology challenge, ranking among the top seven malignancies worldwide with ≈800,000 new cases diagnosed and 400,000 deaths annually.^[^ ^28^ ^]^ The high mortality rate is primarily driven by the recurrent/metastatic (R/M) setting, where median survival drops to 6–9 months despite treatment.^[^ ^29^ ^]^ Although ICIs now constitute first‐line management for R/M HNSCC, over 80% of patients exhibit innate or acquired resistance.^[^ ^30^ ^]^ This profound therapeutic impasse is exacerbated by the absence of reliable predictive biomarkers and the unclear resistance mechanisms. To address this gap, we aimed to identify a biomarker capable of predicting immunotherapy response and serving as a therapeutic target to enhance efficacy. First, we employed four HNSCC ICI‐therapy scRNA‐seq datasets to assess CD8⁺ Tex^term^ cell infiltration dynamics across treatment timepoints (pre‐ vs post‐treatment) and response groups (responders vs non‐responders). Next, we validated these dynamics, predictive efficacy, and association with tumor progression in HNSCC clinical specimens. Finally, we elucidated CD8⁺ Tex^term^ cell differentiation mechanisms using in vitro and xenograft models.

As we know, CD8^+^ T lymphocytes represent the primary effector cells mediating antitumor immune responses, forming the mechanistic foundation for cancer immunotherapy.^[^ ^13^ ^]^ During tumor progression, these cells undergo exhaustion‐driven differentiation into heterogeneous subsets within the TME, broadly categorized as Tex^prog^ and Tex^term^ cells.^[^ ^31^, ^32^, ^33^ ^]^ Our study establishes CD8^+^ Tex^term^ cells as the central orchestrators of immunotherapy failure. Intriguingly, we found that while anti‐PD‐1 therapy reactivated Tex^prog^ cells, it fails to rescue Tex^term^ cells and paradoxically increases their infiltration. This may be due to compensatory TIM‐3/TIGIT upregulation.^[^ ^34^, ^35^, ^36^ ^]^ Consistent with this, high infiltration of CD8⁺ Tex^term^ cells alongside diminished effector CD8⁺ T cells in the HNSCC TME promotes tumor progression and correlates with poor clinical outcomes. Reports indicate that Tex^term^ cells upregulate Treg‐related genes^[^ ^37^ ^]^ and correlate positively with Treg infiltration.^[^ ^38^ ^]^ This aligns with our observation that Tex^term^ cells amplify immunosuppression via CCR5‐dependent Treg recruitment, creating a self‐perpetuating barrier to immunity. The resulting high tolerance mediated by Tex^term^ cells and Tregs fosters an immunosuppressive microenvironment and drives immunotherapy resistance,^[^ ^22^ ^]^ likely contributing to the profoundly immunosuppressive TME in HNSCC.

A primary challenge in cancer immunotherapy lies in the substantial interpatient heterogeneity of treatment sensitivity, with underlying mechanisms remaining elusive. Given the high infiltration of Tex^term^ cells in the HNSCC TME, we investigated their pivotal role in immunotherapy resistance. Notably, recent landmark studies in non‐small cell lung cancer (NSCLC)  link Tex^term^/Treg‐enriched TME subtype to immunotherapy resistance.^[^ ^39^ ^]^ Here, we employed multi‐modal validation across clinical cohorts, scRNA‐seq, and in vivo models to demonstrate that high pre‐treatment stromal Tex^term^ cells density predicts ICIs failure, whereas higher Tex^prog^ cells indicate better response. Considering this, we propose: can the critical role of Tex^term^ cells in immunotherapy resistance be leveraged to predict therapeutic efficacy?

Conventional biomarkers exhibit fundamental limitations: the CPS neglects spatial heterogeneity, and CPS‐negative patients may still respond to aPD‐1 therapy.^[^ ^40^, ^41^ ^]^ While tumor mutational burden (TMB) reflects static genomic metrics rather than dynamic immune evasion.^[^ ^42^ ^]^ Although CD8^+^ Tex^prog^ cells determine immunotherapy efficacy,^[^ ^43^ ^]^ targeting them is limited by their irreversible differentiation into Tex^term^ cells during tumor progression.^[^ ^44^, ^45^ ^]^ In contrast, our study revealed that the stromal Tex^term^ cell density demonstrates superior predictive power (AUC = 0.86). If a patient's tumor biopsy tissue infiltrates a large number of Tex^term^ cells before ICI treatment, it can be predicted that the patient has a poorer treatment effect. This helps clinicians make more precise, personalized decisions about using immunotherapy and improves outcome prediction. Furthermore, we demonstrated that stromal Tex^term^ cells density serves as a prognostic factor for OS and remains independent of two well‐established clinical variables (CPS and p16 status) in HNSCC. These results suggest that the stromal Tex^term^ cells infiltrationcould function as a novel biomarker to identify high‐risk patients beyond conventional biomarkers such as p16 and CPS status.

Recently, Tex^term^ cells have emerged as a critical therapeutic frontier and major research focus in antitumor immunity. Pioneering studies define a four‐stage developmental trajectory for T‐cell exhaustion (Tex^prog1^→Tex^prog2^→Tex^int^→Tex^term^).^[^ ^17^ ^]^ To investigate this trajectory, we performed single‐cell mapping, revealing that tumor‐intrinsic PD‐L1 (not immune cell‐derived PD‐L1^[^ ^44^ ^]^) primarily governs CD8^+^ T exhaustion. Previous studies focused on tumor surface PD‐L1 and local regulation, overlooking systemic immunosuppression by circulating EVs.^[^ ^26^, ^46^ ^]^ Our study further identifies tumor‐derived EVs as master regulators of Tex^term^ cells commitment. We reveal a non‐cell‐autonomous evasion pathway wherein PD‐L1‐enriched EVs^[^ ^26^ ^]^ engage PD‐1 receptors independent of direct cell contact while simultaneously sequestering therapeutic antibodies.^[^ ^47^ ^]^ CRISPR‐mediated PD‐L1 ablation on EVs attenuated Tex^term^ cells accumulation and restored antitumor immunity, establishing EVs as actionable resistance vectors.^[^ ^48^ ^]^ These findings unveil a novel immune evasion axis wherein tumors sustain CD8⁺ T cell exhaustion via continuous PD‐L1⁺ EV release. Critically, aPD‐1 therapy fails to neutralize EV‐mediated PD‐L1/PD‐1 interactions, perpetuating Tex^term^ cells accumulation and immunotherapy resistance. This paradigm underscores the need for dual‐targeting strategies against both membrane‐bound and EV‐associated PD‐L1 to overcome immunotherapy resistance.

The intricate transcriptional network governing T cell exhaustion involves key drivers such as TOX,^[^ ^49^, ^50^ ^]^ Eomes,^[^ ^51^ ^]^ and NR4a.^[^ ^52^ ^]^ Our results identify BATF as a critical inducer of terminal exhaustion in HNSCC. BATF, a member of the activator protein‐1 (AP‐1) transcription factor family, plays a crucial role in CD8^+^ T cell differentiation. Growing evidence indicates that BATF, TOX, and NR4A cooperate within a sustained NFAT‐driven transcriptional network to enforce T cell exhaustion.^[^ ^53^, ^54^, ^55^ ^]^ Under chronic antigen stimulation, the transcriptional complex formed by BATF and IRF4 undergoes functional reprogramming, shifting toward the inhibition of T cell function‐related genes and the promotion of inhibitory receptor (IRs) expression. Concurrently, TOX and NR4A expression increases, synergizing to amplify IR co‐expression and inhibitory signaling.^[^ ^56^ ^]^ Within this network, BATF not only intensifies the exhaustion programs mediated by TOX and NR4A but also may form a synergistic and self‐reinforcing transcriptional module that drives terminal T cell exhaustion. Although BATF is known to regulate effector memory differentiation in CD8^+^ T cells^[^ ^53^ ^]^ and has been implicated in CAR‐T cell dysfunction by upregulating exhaustion‐associated genes,^[^ ^54^ ^]^ its upstream regulators remained unclear. To the best of our knowledge, we provide the first demonstration that PD‐L1⁺ EVs upregulate BATF expression in CD8⁺ T cells, thereby driving terminal exhaustion—an effect reversed by PD‐L1 depletion on EVs. This finding underscores a close relationship between PD‐L1/PD‐1 pathway activation and BATF upregulation. The potential molecular link between PD‐1/PD‐L1 signaling and BATF upregulation whereby PD‐1 engagement recruits phosphatases such as SHP2, modulating downstream signaling cascades that lead to BATF induction.^[^ ^57^ ^]^ As a key transcriptional regulator, BATF suppresses T cell function by inhibiting proliferation and cytokine secretion.^[^ ^58^, ^59^ ^]^ Our results suggest that PD‐L1^+^ EVs similarly trigger this pathway through activation of PD‐1 signaling in CD8^+^ T cells, resulting in BATF upregulation and terminal exhaustion. This alignment with the canonical PD‐1/BATF axis reinforces the robustness of our findings and suggests that targeting BATF could be a viable strategy to overcome EV‐mediated immune evasion in HNSCC.

A critical aspect of EV research is the heterogeneity of isolated vesicles, since different subtypes (e.g., exosomes, microvesicles) can exhibit distinct biological activities. In this study, EVs were enriched from HNSCC cell‐conditioned media using differential centrifugation and ultracentrifugation—a standard method for exosomes isolation.^[^ ^60^ ^]^ Furthermore, nanoparticle tracking analysis from our previous study^[^ ^11^ ^]^ confirmed that the isolated particles from SNU1076 and SCC7 cells fell predominantly within the exosomal size range, with a negligible proportion exceeding 200 nm (typically indicative of microvesicles). Thus, we conclude that the observed induction of BATF‐driven terminal exhaustion in CD8⁺ T cells is primarily mediated by exosomes. That said, we cannot entirely rule out minor contributions from other low‐abundance EV subtypes. Further studies applying more refined approaches, such as immunoaffinity capture targeting exosome‐specific markers (e.g., CD63, CD81), could help definitively attribute this immunomodulatory function to a specific exosomal population.

**Figure 9:**
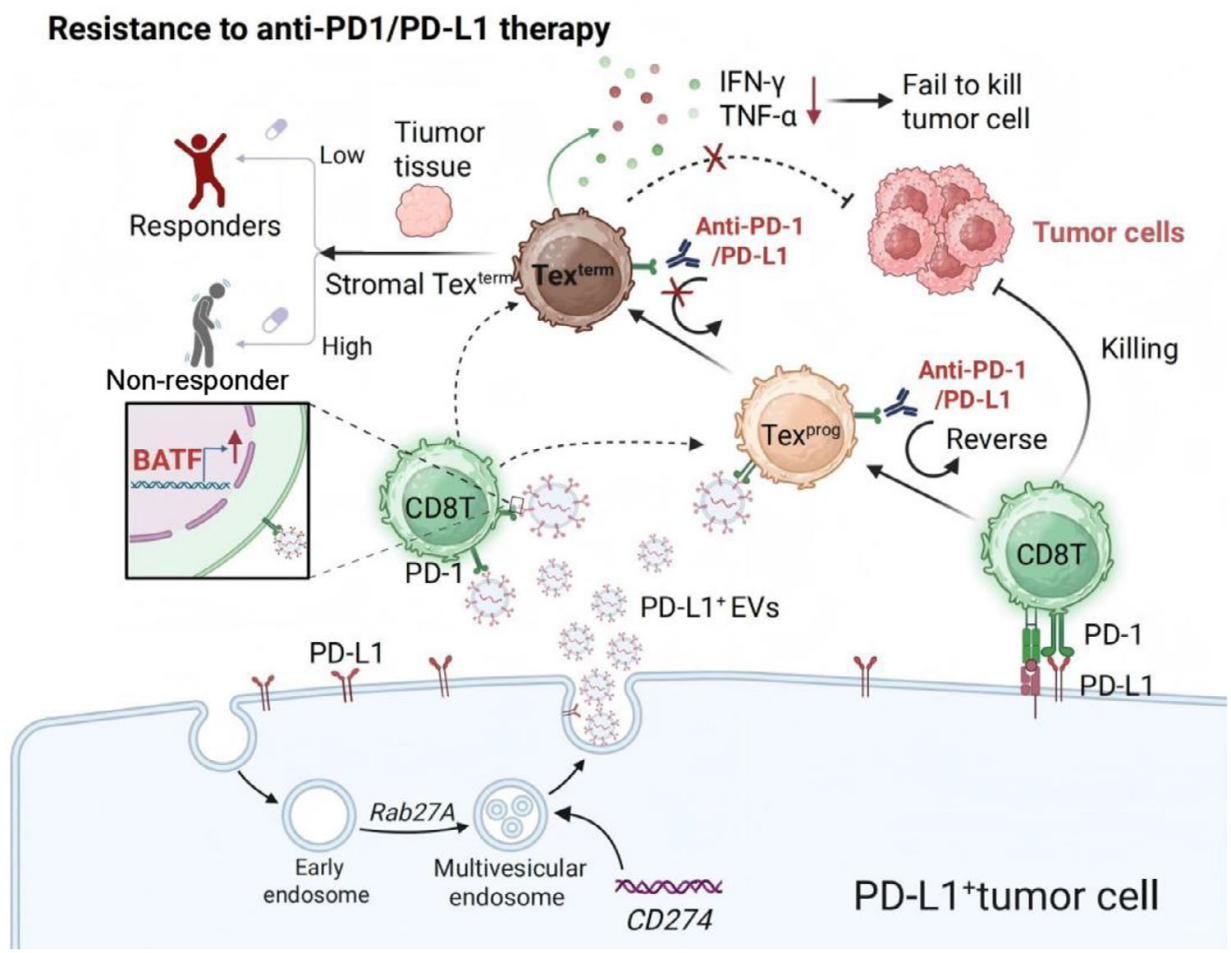
Mechanisms of resistance to anti‐PD‐1 therapy mediated by tumor‐derived PD‐L1^+^ extracellular vesicles (EVs) and the predictive role of stromal terminal exhausted T (Tex^term^) cells for the patient's response to immunotherapy. Tex^prog^ cells, precursor exhausted T cells. Created in https://BioRender.com/mo469ce.

Collectively, our findings illuminate the EV‐PD‐L1‐BATF‐Tex^term^ axis as the mechanistic cornerstone of immunotherapy resistance in HNSCC (**Figure** **9**). Currently, inhibition of EV secretion, such as GW4869 (nSMase2 inhibitor), tipifarnib (farnesyltransferase inhibitor), and neviramine (Rab27a inhibitor), has demonstrated efficacy in suppressing tumor‐derived EVs release, thereby attenuating immune evasion in preclinical models.^[^ ^61^, ^62^ ^]^ Several of these agents are currently under evaluation in early‐phase clinical trials in combination with immunotherapies.^[^ ^63^, ^64^ ^]^ The application of high‐affinity neutralizing agents such as anti‐PD‐L1 agents, including atezolizumab and durvalumab, is already widely used in clinical practice. However, these approaches have not yet achieved consistently precise therapeutic outcomes. Recently, the emergence of PD‐L1 nanobodies^[^ ^65^, ^66^ ^]^ has provided new hope for precisely targeting PD‐L1^+^EVs, but it is still in the preclinical research exploration stage. Future studies should focus on combining tumor‐selective EV inhibitors, anti‐PD‐L1 nanobodies, and BATF inhibition—a strategy with strong preclinical rationale that may synergistically restore T cell function and counteract EV‐mediated immunosuppression, offering a clinically actionable approach to improve responses to immune checkpoint inhibitor therapy.

## 4 Experimental Section

### Patient Cohort and Sample Collection

This study recruited 34 patients with newly diagnosed HNSCC who underwent surgical resection at the First Affiliated Hospital of Sun Yat‐sen University. A total of 34 fresh tumor tissues and paracarcinoma tissues were collected during surgical resection. The basic information of the patients is shown in Table S12 (Supporting Information). Peripheral blood samples were obtained from 27 patients with HNSCC and 21 healthy volunteers. The tissues and blood samples were subsequently processed into single‐cell suspensions for flow cytometry analysis.

A total of 40 HNSCC patients who received Keytruda (K) monotherapy or K combined with chemotherapy (Taxol and platinum, TP) for neoadjuvant or adjuvant treatment at the First Affiliated Hospital of Sun Yat‐sen University between December 2020 and June 2023 were retrospectively enrolled. Pretreatment biopsy tissue sections from these patients were collected for mIHC analysis. The immunotherapy response of these patients was subsequently evaluated using the Response Evaluation Criteria in Solid Tumors version 1.1 (RECIST 1.1) scoring system. Additionally, follow‐up information, including OS and PFS data, was collected. The follow‐up timeline concluded on May 30, 2024. The tumor–node–metastasis (TNM) clinical stages of patients were determined based on the eighth edition of the American Joint Committee on Cancer Tumor, Node, and Metastasis System. The details for these 40 patients are shown in Table S13 (Supporting Information).

Using scRNA‐seq, six primary HNSCC tissue samples and two adjacent normal mucosa specimens were analyzed, as described in our prior study.^[^ ^10^ ^]^ Additionally, publicly available scRNA‐seq datasets were incorporated from the Gene Expression Omnibus (GEO) database (https://www.ncbi.nlm.nih.gov/geo/) under accession codes GSE234933, GSE195832, and GSE200996 for further analysis. Pretreatment scRNA‐seq data for 9 patients with HNSCC who were resistant to PD‐1 treatment (non‐responder group, NR) and 5 patients who responded positively to the treatment (responder group, R) were included in the GSE234933 dataset^[^ ^67^ ^]^ and assessed in this study. Moreover, data on 8 samples from the GSE195832 dataset and 25 samples from the GSE200996 dataset were also assessed in this study. These samples were collected from patient tumors before and after treatment in a trial of HNSCC patients who were treated with the aPD‐1 therapy nivolumab.^[^ ^68^, ^69^ ^]^

### Statistical Analysis of scRNA‐Seq Data

The analysis of the scRNA‐seq data was performed via the NovelBrain Cloud Analysis Platform (www.novelbrain.com). Fastp (version 0.20.1, https://github.com/OpenGene/fastp) was utilized with default settings to filter adaptor sequences and remove low‐quality and short reads, resulting in clean data.^[^ ^70^ ^]^ Subsequently, feature‒barcode matrices were generated by aligning reads to the human genome (GRCh38 Ensembl, version 91) using CellRanger v3.1.0. The optimal cell parameters were determined while excluding cells with counts equal to 10000, considering UMI counts and Cell‐UMI slopes. Cells expressing between 200 and 500 genes, with mitochondrial UMI rates less than 20%, were retained after quality filtering, and mitochondrial genes were excluded from the expression table.

For normalization and regression, the Seurat package (version 3.1.4, https://satijalab.org/seurat/)^[^ ^71^ ^]^ was employed to analyze UMI counts and mitochondrial percentages to yield scaled data. Principal component analysis (PCA) was performed on the top 2000 highly variable genes, and the top 10 principal components were subsequently used for UMAP visualization. Cell types were defined based on the established biomarker information. To subcluster each cell type, it was noted that the canonical correlation analysis (CCA) algorithm was less effective in addressing batch effects; thus, the mutual nearest neighbors (MNN) algorithm was applied from the scran package (http://www.bioconductor.org/packages/release/bioc/html/scran.html) with a k value of 5 for batch effect correction. Following the MNN analysis, graph‐based clustering was performed using optimized parameters, complemented by marker analysis for accurate cell identification, and the clusters were detected with a resolution of 0.8.

Gene Ontology (GO) analysis was conducted to investigate the differentially expressed genes according to annotations sourced from the NCBI (http://www.ncbi.nlm.nih.gov/), UniProt (http://www.uniprot.org/), and GO (http://www.geneontology.org/) websites. A cell communication analysis utilizing CellPhoneDB^[^ ^72^ ^]^ was conducted, a publicly available database of ligands, receptors, and their interactions, to elucidate cellular interactions.

In the GSE234933 cohort, the HN8 sample with no CD8^+^ T cells was excluded, and the remaining 13 samples were grouped based on their PD‐L1 expression on cancer cells. To compare the gene expression profiles between PD‐L1^+^ tumors and PD‐L1^−^ tumors, findMarkers was used to identify differentially expressed genes (DEGs) using the default parameters.

### The Cancer Genome Atlas (TCGA) Analysis

To assess the prognostic impact of the signature gene sets derived from PD‐L1^+^ EVs and Tex^term^ cells, the gene expression profiles and clinical data of HNSCC patients were analyzed and were obtained from the TCGA data portal (https://portal.gdc.cancer.gov/cart). This study focused on examining the relationships among PD‐L1^+^ EVs signature, Tex^term^ signature, *BATF*, and *CD279* mRNA expression, and their clinical prognostic value. For all survival analyses, patients were categorized into high‐ and low‐expression groups based on the optimal cutoff value. Kaplan–Meier survival curves were generated using R‐4.0.3 with the survminer package.^[^ ^73^ ^]^ Prognosis‐associated genes in HNSCC were analyzed using the TCGA database.

### Cell Lines and Isolation of CD8^+^ T Cells

The human HNSCC cell line SNU1076 (RRID: CVCL_5006) purchased from Meisen Cell Biotechnology Co., Ltd. (Hangzhou, China) was used in this study. The murine HNSCC cell line SCC7 (RRID: CVCL_V412), syngeneic to C3H mice, was generously provided by the Otolaryngology Research Laboratory at the Sixth Affiliated Hospital of Sun Yat‐sen University.^[^ ^74^ ^]^ The cell lines SNU1076 and SCC7 were authenticated by short tandem repeat (STR) profiling within the past 12 months and confirmed to be free of mycoplasma contamination. The SNU1076‐PD‐L1^KO^ and SCC7‐PD‐L1^KO^ cell lines were constructed using CRISPR‐Cas9 technology, as previously reported.^[^ ^26^ ^]^ Cells were cultured in DMEM medium.

Peripheral blood mononuclear cells (PBMCs) were extracted from the peripheral blood of healthy volunteers using density gradient centrifugation. CD8^+^ T cells were subsequently isolated from PBMCs using flow cytometry sorting technology. The cells were maintained in RPMI 1640 or DMEM supplemented with 10% FBS (Gibco) or exosome‐free FBS (ViVaCell, BI) and 1% penicillin‒streptomycin (Gibco, #15070063).

Enzymatic digestion of tumor tissues and adjacent normal nontumor tissues was conducted using a collagenase/hyaluronidase mixture (Stemcell, #0792) in a solution comprising 90% DMEM, 5% FBS, and 1 mg mL^−1^ DNase I. The resulting single‐cell suspension was filtered through a 70 µm nylon filter (BD Biosciences). CD8^+^ T cells were obtained from the tissue single‐cell suspension through magnetic bead sorting.

### Flow Cytometry

After red blood cell lysis, the cells were washed twice with cold PBS and pre‐stimulated with a cell stimulation cocktail (eBioscience, #TNB‐4975) for 6 h. The cells were then labeled in complete darkness at 4 °C for 30 min with a panel of fluorescently conjugated antibodies, including a near‐IR reactive dye (Invitrogen, #2339908), anti‐hCD45 (BioLegend, #368549), anti‐mCD45 (BioLegend, #103138), anti‐hCD3 (BioLegend, #317340), anti‐mCD3 (BioLegend, #100260), anti‐hCD8 (BioLegend, #344712), anti‐mCD8 (BioLegend, #100740), anti‐hPD‐1 (BioLegend, #329739), anti‐mPD‐1 (BioLegend, #135231), anti‐hTIGIT (BioLegend, #372744), anti‐mTIGIT (BioLegend, #142110), anti‐hTIM‐3 (BD, #345032), anti‐mTIM‐3 (BD, #747618), anti‐TCF‐1 (BD, #564217) and anti‐hCD226 (BioLegend, #338334). Next, the cells were permeabilized using the Foxp3/Transcription Factor Staining Kit (eBioscience, #00‐5523‐00) and incubated with anti‐TCF‐1 (BioLegend, #100412), anti‐hIFN‐γ (BD, #563563), anti‐hTNF‐α (BD, #551384) and anti‐hBATF (BD, 46‐9860‐42) antibodies for nuclear staining in the dark at 4 °C. Finally, the cells were washed and resuspended for analysis.

### Multiplex Immunofluorescence

Formalin‐fixed, paraffin‐embedded (FFPE) tissue sections (4 µm) were deparaffinized and rehydrated. Antigen retrieval was performed using Tris‐EDTA buffer, followed by peroxidase blocking at room temperature (RT). The slides were incubated with primary antibodies (CD8, Abcam, 1:1000; PD‐1, Abcam, 1:2000; TCF‐1, CST, 1:1000; TIM‐3, CST, 1:500; Pan‐CK, Abcam, 1:1000) overnight at 4 °C and then with HRP‐conjugated secondary antibodies (BioMed World, WAS12011) at RT. After being washed with 1× PBS, the slides were incubated with fluorophore‐conjugated tyramide signal amplification (TSA) (TSA570, WAS10031; TSA520, WAS10021; TSA620, WAS10041; TSA690, WAS10061) at RT. Microwave treatment was applied to strip the antibody complexes before they were labeled with the next marker. The final staining step included DAPI staining at room temperature and mounting with anti‐fade medium. Images were captured using a PANNORAMIC MIDI II slide scanner (3DHISTECH). Carlson Tsui et al.^[^ ^75^ ^]^ characterized the precursors of exhausted T (Tex^prog^) cells as PD‐1^+^TCF‐1^+^TIM‐3^−^CD8^+^ T cells and Tex^term^ cell as PD‐1^+^TCF‐1^−^TIM‐3^+^CD8^+^ T cells.^[^ ^76^ ^]^ Optimal cutoff values were established using the ggsurvplot R package to divide the samples into high‐ and low‐expression groups. The tumor areas, stromal areas, and TME were histologically analyzed, and the proportions of total CD8^+^ T cells in the tumor and stromal areas were assessed.

### Extracellular Vesicle Isolation and CRISPR‐Cas9 Gene Knockout

EVs were isolated from cell culture supernatant by differential centrifugation and ultracentrifugation, as previously described,^[^ ^26^ ^]^ with the following key steps: Supernatant was first centrifuged at 300 × g for 10 min at 4 °C to remove intact cells, followed by centrifugation at 2000 × g for 20 min at 4 °C to pellet dead cells and large debris. The supernatant was then subjected to high‐speed centrifugation at 10 000 × g for 30 min at 4 °C to further clear cellular debris. EVs were pelleted via ultracentrifugation at 100 000 × g for 70 min at 4 °C. The resulting EV pellet was washed in phosphate‐buffered saline (PBS) and ultracentrifuged again under the same conditions (100 000 × g, 70 min, 4 °C) to obtain purified EVs. The final pellet was resuspended in 100 µL of PBS and stored at −80 °C. EVs identity and purity were confirmed using nanoparticle tracking analysis (NTA) and transmission electron microscopy (TEM).

Stable PD‐L1 knockout (PD‐L1^KO^) cell lines were generated from SNU1076 and SCC7 cells using CRISPR‐Cas9 technology. The absence of PD‐L1 expression in isolated PD‐L1^KO^EVs was verified by western blot analysis, as reported in our previous study.^[^ ^26^ ^]^

### In Vitro Coculture Assay

For in vitro experiments, 8*10^5^ CD8^+^ T cells were cultured in a 48‐well plate with EV‐free complete medium supplemented with 5 µg mL^−1^ anti‐CD3 (eBioscience, #16‐0037‐85)/2.5 µg mL^−1^ anti‐CD28 (eBioscience, #16‐0289‐85) antibodies and 25 ng mL^−1^ IL‐2 (PeproTech, #200‐02) for 5 days. The experimental groups were subsequently treated with PD‐L1^+^ EVs or PD‐L1^KO^ EVs (6×10^8^ particles), whereas the control group received PBS for an additional 3 days. Flow cytometry was used to assess CD8^+^ T cell exhaustion.

SMART‐seq was conducted on harvested cells from the treatment group (n = 18 samples), which included PBS, PD‐L1^+^ EVs, and PD‐L1^KO^ EVs, by Lianchuan Biological Co. This analysis aimed to evaluate the impact of these treatments on immune function. Genes with |log2(fold change)| > 1 and *P* value < 0.05 were classified as DEGs. These DEGs were subjected to Kyoto Encyclopedia of genes and genomes (KEGG) enrichment analysis. Gene set enrichment analysis (GSEA) was subsequently performed using the GSEA function from the cluster profiler package (version 4.10.1), with a focus on the hallmark gene set collection to identify significantly enriched pathways based on the KEGG pathway analysis.

### Murine Subcutaneous Xenograft Model and Therapeutic Interventions

The immunocompetent female wild‐type C3HeN mice,^[^ ^77^ ^]^ 6–8 weeks old, weighing 15–20 g, were selected and housed in a sterile, specific‐pathogen‐free (SPF) environment at the Animal Experiment Center of Sun Yat‐sen University. To establish subcutaneous xenograft models of HNSCC, 1*10^6^ wild‐type SCC7 or SCC7‐PD‐L1^KO^ cells were harvested and injected subcutaneously into the flank of each mouse in a volume of 100 µL of PBS. When the tumor size reached ≈50 mm^3^, the mice were treated via the tail vein with PD‐L1^+^ EVs or PD‐L1^KO^ EVs (80 µg in 200 µL of PBS per mouse for each injection) twice weekly for two weeks. For in vivo antibody treatment, the mice received intraperitoneal injections of either 200 µg of the IgG1 isotype control (BioXCell, #BE0083) or aPD‐1 antibodies (BioXCell, #BE0146) every three days for two weeks. Tumor growth curves were generated. At the end of the study, tumor tissue, spleen, and lymph nodes were harvested from the mice for flow cytometry analysis to assess the impact of the treatments on the tumor microenvironment.

### Statistical Analysis

All the statistical analyses were performed using IBM SPSS Statistics (V.23) or GraphPad Prism V.10 software. Prior to analysis, the quantitative data were subjected to normality testing and variance equality assessments. The data are presented as the means ± standard errors of the means (SEMs) or medians (interquartile ranges). Statistical comparisons were made using two‐tailed t tests, one‐way analysis of variance (ANOVA), or two‐way ANOVA in GraphPad Prism 5 (RRID: SCR_002798) or R 3.6.0 software (RRID: SCR_001905), followed by Bonferroni's multiple comparisons test. For survival analyses, Kaplan‒Meier plots were generated, and significant differences were assessed using the log‐rank test. To evaluate the relationships between two factors, pearson correlation analysis was utilized. Differences were deemed statistically significant at a *P* value of less than 0.05.

### Ethics Approval and Consent to Participate

This study was approved by the Ethics Committee of the First Affiliated Hospital, Sun Yat‐sen University (Approval Nos:[2023]283 and [2023]787). The animal studies were performed after the approval of Sun Yat‐sen University's Laboratory Animal Care and Use Committee (Approval Nos: SYSU‐IACUC‐2021000515 and 2023002788).

## Conflict of Interest

The authors declare no conflict of interest.

## Author Contributions

R.H.F., B.X.H., and Y.L. contributed equally to this work. R.H.F., B.X.H., and Y.L. designed and performed the experiments, analyzed data, and wrote the manuscript; Z.S.C., J.H.Z., S.Y.L., Z.L.X., and X.Q.C. performed experiments and analyzed data; W.S., Y.L., and Y.C.D. provided patient tissue samples. Z.C., and W.B.G. provided critical suggestions and technical support; W.P.W., W.B.L., and K.X.L. supervised this project, designed the experiments, and reviewed the manuscript. All authors read and approved the final manuscript.

## Supporting information



Supplemental Table 1

Supplemental Table 2

Supplemental Table 3

Supplemental Table 4

Supplemental Table 5

Supplemental Table 6

Supplemental Table 7

Supplemental Table 8

Supplemental Table 9

Supplemental Table 10

Supplemental Table 11

Supplemental Table 12

Supplemental Table 13

Supporting Information

Supporting Information

## Data Availability

Data are available in a public, open‐access repository. Data are available upon reasonable request. The TCGA‐HNSCC data set is accessible via the National Cancer Institute Genomic Data Commons Data Portal (https://portal.gdc.cancer.gov/). The single‐cell RNA sequencing data set can be accessed via Gene Expression Omnibus (GEO) with the accession numbers GSE234933, GSE195832, and GSE200996. Data from the HNSCC patient cohort are available upon reasonable request.
