# Supplementary material for: PD‐L1 on Tumor‐Derived Extracellular Vesicles Induces CD8+ T Cell Terminal Exhaustion and Mediates Anti‐PD‐1 Resistance in Head and Neck Squamous Cell Carcinoma
Source: Adv Sci (Weinh). 2025 Nov 5;13(4):e16348. doi: 10.1002/advs.202516348 (PMC12822461; doi:10.1002/advs.202516348)
Supplement: Supplementary file 12 — Supplemental Table 12 [file ADVS-13-e16348-s006.docx]

**Table. S1 Basic information of 34 patients with HNSCC .**

| No. | Gender | Tumor location | T | N | M | Clinical stage (TNM) |
| --- | --- | --- | --- | --- | --- | --- |
| 1 | Male | Oropharynx | 2 | 0 | 0 | II |
| 2 | Male | Larynx | 4a | 1 | 0 | IVA |
| 3 | Male | Larynx | 3 | 0 | 0 | III |
| 4 | Male | Larynx | 3 | 0 | 0 | III |
| 5 | Male | Larynx | 3 | 0 | 0 | III |
| 6 | Male | Larynx | 3 | 0 | 0 | III |
| 7 | Male | Larynx | 4a | 1 | 0 | IVA |
| 8 | Male | Larynx | 4 | 1 | 0 | IVA |
| 9 | Male | Larynx | 2 | 0 | 0 | II |
| 10 | Male | Larynx | 3 | 0 | 0 | III |
| 11 | Male | Larynx | 2 | 1 | 0 | II |
| 12 | Male | Larynx | 3 | 0 | 0 | III |
| 13 | Male | Larynx | 3 | 0 | 0 | III |
| 14 | Female | Hypoparynx | 2 | 0 | 0 | II |
| 15 | Male | Larynx | 4a | 2 | 0 | IVA |
| 16 | Male | Larynx | 3 | 2 | 0 | III |
| 17 | Male | Larynx | 4a | 0 | 0 | IVA |
| 18 | Male | Larynx | 1 | 0 | 0 | I |
| 19 | Male | Larynx | 3 | 0 | 0 | III |
| 20 | Male | Larynx | 3 | 2c | 0 | IVA |
| 21 | Male | Hypoparynx | 3 | 0 | 0 | III |
| 22 | Male | Larynx | 4A | 2 | 0 | IVB |
| 23 | Male | Larynx | 1 | 0 | 0 | I |
| 24 | Male | Larynx | 4b | 3 | 1 | IVB |
| 25 | Male | Larynx | 4a | 0 | 0 | IVA |
| 26 | Male | Larynx | T3 | 0 | 0 | III |
| 27 | Male | Larynx | 4a | 0 | 0 | IVA |
| 28 | Male | Larynx | 4a | 0 | 0 | IV |
| 29 | Male | Larynx | 2 | 2 | 0 | IVA |
| 30 | Male | Larynx | 3 | 0 | 0 | III |
| 31 | Male | Larynx | 3 | 0 | 0 | III |
| 32 | Male | Larynx | 1b | 0 | 0 | I |
| 33 | Male | Larynx | 3 | 0 | 0 | III |
| 34 | Male | Oropharynx | 4 | 0 | 0 | IVA |

Abbreviations: TNM, tumor, node, metastasis.
