# Supplementary material for: PD‐L1 on Tumor‐Derived Extracellular Vesicles Induces CD8+ T Cell Terminal Exhaustion and Mediates Anti‐PD‐1 Resistance in Head and Neck Squamous Cell Carcinoma
Source: Adv Sci (Weinh). 2025 Nov 5;13(4):e16348. doi: 10.1002/advs.202516348 (PMC12822461; doi:10.1002/advs.202516348)
Supplement: Supplementary file 13 — Supplemental Table 13 [file ADVS-13-e16348-s002.docx]

**Table. S2 Basic information and treatment outcomes about 40 patients with HNSCC who received immunochemotherapy as Initial therapy.**

| No. | Gender | Age | Tumor location | Smoking | Driking | Clinical stage | Treatment option | Outcome |
| --- | --- | --- | --- | --- | --- | --- | --- | --- |
| 1 | Male | 66 | Hypoparynx | Yes | No | ⅣA | K+TP | PR |
| 2 | Male | 53 | More than one site | Yes | Yes | ⅣA | K+TP | PR |
| 3 | Male | 64 | Hypoparynx | No | No | ⅣA | K+TP | PR |
| 4 | Male | 50 | Oropharynx or others | No | No | III | K+TP | PR |
| 5 | Male | 55 | Larynx | Yes | No | R/M | K+TP | SD |
| 6 | Male | 43 | More than one site | No | No | R/M | K+TP | PR |
| 7 | Male | 55 | Oropharynx or others | Yes | No | ⅣA | K+TP | CR |
| 8 | Male | 74 | Hypoparynx | Yes | Yes | R/M | K | PR |
| 9 | Male | 65 | More than one site | Yes | Yes | III | K+TP | PR |
| 10 | Male | 65 | Hypoparynx | No | No | ⅣA | K+TP | SD |
| 11 | Male | 57 | Oropharynx or others | No | No | Ⅰ | K+TP | PR |
| 12 | Male | 64 | Hypoparynx | No | Yes | ⅣA | K+TP | SD |
| 13 | Male | 66 | Hypoparynx | Yes | Yes | III | K | PD |
| 14 | Male | 86 | Hypoparynx | No | No | IVB | K | PR |
| 15 | Male | 52 | More than one site | Yes | Yes | III | K+TP | CR |
| 16 | Male | 71 | Oropharynx or others | Yes | Yes | R/M | K+TP | PR |
| 17 | Male | 56 | Hypoparynx | Yes | Yes | IVB | K+TP | PR |
| 18 | Male | 62 | Hypoparynx | No | Yes | ⅣA | K+TP | PR |
| 19 | Male | 65 | Larynx | Yes | Yes | R/M | K+TP | PR |
| 20 | Male | 57 | Oropharynx or others | Yes | No | Ⅱ | K+TP | PD |
| 21 | Male | 77 | Larynx | Yes | No | R/M | K | SD |
| 22 | Male | 63 | Hypoparynx | Yes | Yes | ⅣA | K | SD |
| 23 | Male | 58 | Hypoparynx | No | Yes | III | K+TP | PR |
| 24 | Male | 81 | Hypoparynx | Yes | Yes | Ⅱ | K+TP | SD |
| 25 | Male | 49 | More than one site | Yes | Yes | ⅣA | K+TP | SD |
| 26 | Female | 47 | Oropharynx or others | No | No | III | K | PD |
| 27 | Male | 63 | Hypoparynx | No | No | ⅣA | K+TP | PR |
| 28 | Male | 54 | Hypoparynx | Yes | No | ⅣA | K+TP | PR |
| 29 | Male | 78 | Hypoparynx | Yes | No | ⅣA | K+TP | PR |
| 30 | Male | 75 | Larynx | Yes | Yes | R/M | K+TP | SD |
| 31 | Male | 62 | Oropharynx or others | Yes | Yes | III | K | SD |
| 32 | Male | 66 | More than one site | Yes | Yes | ⅣA | K+TP | PR |
| 33 | Male | 46 | Hypoparynx | Yes | Yes | ⅣA | K+TP | PR |
| 34 | Male | 56 | Oropharynx or others | Yes | Yes | III | K+TP | PR |
| 35 | Male | 29 | Oropharynx or others | No | No | ⅣA | K+TP | PR |
| 36 | Male | 63 | Hypoparynx | No | No | Ⅱ | K+TP | SD |
| 37 | Male | 49 | Hypoparynx | Yes | Yes | ⅣA | K+TP | PR |
| 38 | Male | 59 | Hypoparynx | Yes | Yes | ⅣA | K+TP | PR |
| 39 | Male | 57 | More than one site | Yes | Yes | R/M | K | PD |
| 40 | Male | 64 | Hypoparynx | Yes | Yes | R/M | K+TP | PR |

Abbreviations: PR, partial response; CR, complete response; PD, progressive disease; SD, stable disease; R/M, recurrent or metastatic; TP, Taxel + Cisplatin; K, keytruda.
