# Supplementary material for: PD‐L1 on Tumor‐Derived Extracellular Vesicles Induces CD8+ T Cell Terminal Exhaustion and Mediates Anti‐PD‐1 Resistance in Head and Neck Squamous Cell Carcinoma
Source: Adv Sci (Weinh). 2025 Nov 5;13(4):e16348. doi: 10.1002/advs.202516348 (PMC12822461; doi:10.1002/advs.202516348)
Supplement: Supplementary file 14 — Supporting Information [file ADVS-13-e16348-s004.zip › SNU1076 STR.pdf]

Cell Line Authentication Service
STR Profile Report

**Sample Submitted By:**	Dr. Zuokang Zheng<br>Zhejiang Meisen Cell Technology Co.,Ltd.
**Email Address:**	947638289@qq.com
**Sales Order:**	210715B
**Cell Line Designation:**	SNU-1076
**Date Sample Received:**	Jul 15th, 2021
**Report Date:**	Jul 15th, 2021

Methodology: Nineteen short tandem repeat (STR) loci plus the gender determining locus, Amelogenin, were amplified using the commercially available EX20 Kit from AGCU. The cell line sample was processed using the ABI Prism® 3130 XL Genetic Analyzer. Data were analyzed using GeneMapper® ID v3.2 software (Applied Biosystems). Appropriate positive and negative controls were run and confirmed for each sample submitted.

Data Interpretation: Cell lines were authenticated using Short Tandem Repeat (STR) analysis as described in 2012 in ANSI Standard (ASN-0002) by the ATCC Standards Development Organization (SDO) and in Capes-Davis et al., Match criteria for human cell line authentication: Where do we draw the line? Int J Cancer. 2013;132(11):2510-9.

GTB™ performs STR Profiling following ISO 9001:2008 and ISO/IEC 17025:2005 quality standards.
There are no warranties with respect to the services or results supplied, express or implied, including, without limitation, any implied warranty of merchantability or fitness for a particular purpose. Genetic Testing Biotechnology (GTB) is not liable for any damages or injuries resulting from receipt and/or improper, inappropriate, negligent or other wrongful use of the test results supplied, and/or from misidentification, misrepresentation, or lack of accuracy of those results. Your exclusive remedy against GTB and those supplying materials used in the services for any losses or damage of any kind whatsoever, whether in contract, tort, or otherwise, shall be, at GTB's option, refund of the fee paid for such service or repeat of the service.

NOTE: According to the recommendations of *IJC* on cell line authentication, the report is valid for 3 years since the issue date.

Technical Questions?
GTB Technical Support
+86-512-67486171
service@jsdna.org
Section 505, Yixin BLD
SIP, Suzhou, 215123
Jiangsu, P.R. China

Ordering Questions?
order@jsdna.org
GTB Corporation
+86-512-62806339
Section 303, Yixin BLD
SIP, Suzhou, 215123
Jiangsu, P.R. China

GENETIC TESTING BIOTECHNOLOGY

Cell Line Authentication Service
STR Profile Report

Sales Order: 210715B

Loci	Test Results for Submitted Sample<br>**Query Profile:** SNU-1076		ExPASy Reference Database Profile<br>**Database Profile:** SNU-1076	
Amelogenin	X	Y	X	Y
D3S1358	15			
D13S317	8	10	8	10
D7S820	11	12	11	12
D16S539	13		13	
Penta E	12	22		
TPOX	8	11	8	11
TH01	6		6	
D2S1338	19	24		
CSF1PO	11	12	11	12
Penta D	9	10		
D19S433	15.2			
vWA	16		16	
D21S11	30			
D18S51	14			
D6S1043	13	20		
D8S1179	10	15		
D5S818	9	12	9	12
D12S391	19	20		
FGA	24	25		

The allele match algorithm compares the 8 core loci plus amelogenin only, even though alleles from all loci will be reported when available.

Note: Loci highlighted in grey (8 core STR loci plus Amelogenin) can be made public to verify cell identity. In order to protect the identity of the donor, ***please do not publish*** *the allele calls from all the STR loci tested. The sample match is based on the reference data available at the time of comparison.*

Explanation of Test Results

Cell lines with ≥80% match are considered to be related; i.e., derived from a common ancestry. Cell lines with between a 55% to 80% match require further profiling for authentication of relatedness.

☐ The submitted sample profile is human, but not a match for any profile in the ExPASy STR database.

☑ The submitted profile is an exact match for the following human cell line(s) in the ExPASy STR database (8 core loci plus Amelogenin): SNU-1076

☐ The submitted profile is similar to the following ExPASy human cell line(s):

e-Signature, Technician: Digitally signed by Xuekun Chen
DN: cn=Xuekun Chen, o=Genetic Testing Biotechnology (Suzhou), ou=DNA Typing Section, email=order@jsdna.org, c=CN
Date: 2021.07.15 17:22:41 +08'00'

GENETIC TESTING BIOTECHNOLOGY
GUARANTEE
CELL LINE AUTHENTICATION
★ ★ ★ ★ ★

e-Signature, Reviewer: Digitally signed by Xiankun Zhao
DN: cn=Xiankun Zhao, o=Genetic Testing Biotechnology (Suzhou), ou=Supervision Section, email=service@jsdna.org, c=CN
Date: 2021.07.15 17:39:05 +08'00'

Addendum: Electropherogram for the customer's sample set 1 of 1

Cell Line Authentication Service
STR Profile Report

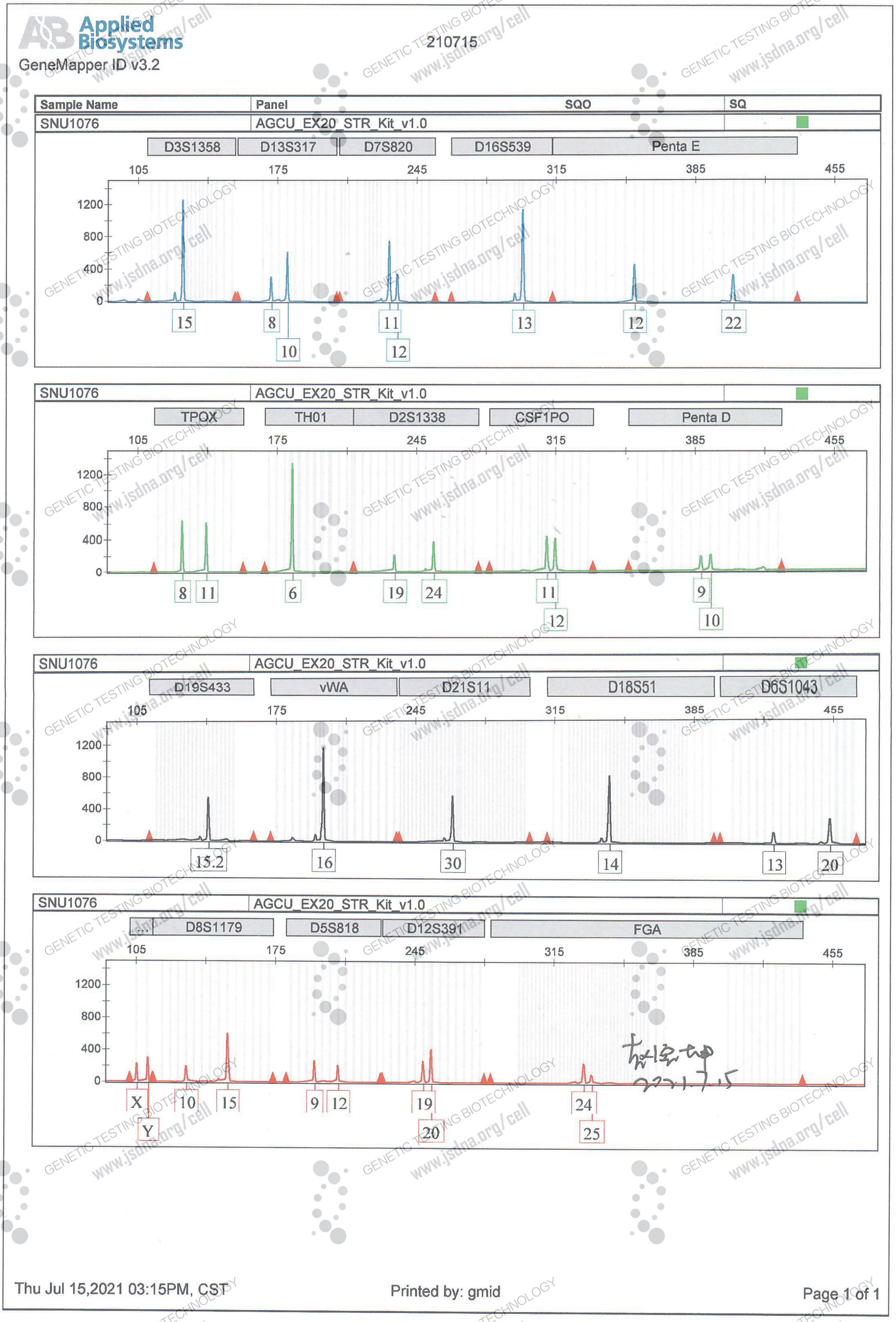

GENETIC TESTING BIOTECHNOLOGY www.jsdna.org/cell

Accession	Name	Nº Markers	Score	Amel	CSF1PO	D2S1338	D3S1358	D5S818	D7S820	D8S1179	D13S317	D16S539	D18S51	D19S433	D21S11	FGA	Penta D	Penta E	TH01	TPOX	vWA
NA	Query	NA	NA	X,Y	11,12			9,12	11,12		8,10	13							6	8,11	16
CVCL_5006 *Best*	SNU-1076	9	100.00%	X,Y	11,12	19,24	15	9,12	11,12	10,15	8,10	13	14	15.2	30	24,25	9,10	12,22	6	8,11	16
CVCL_5006 *Worst*	SNU-1076	9	93.75%	X,Y	11,12	19,24	15	9,12	11,12	10,15	8,10	13	14	15.2	30	24,25	9,10	12,22	6,7	8,11	15,16
CVCL_3047	NCC-RbC-39	9	80.00%	X,Y	12			9,10	11,12		8,10	12							6	8,11	16,17
CVCL_0835	Hs 674.Sk	9	77.42%	X,Y	9,11			12	11,12		10	12,13							6,9.3	8,11	16,19
CVCL_2658	NO36	9	77.42%	X,Y	11,12	25	15,16	12,13	11	12	8	11,13	21,22	15,16	28,29	22			6,7	8,11	16,18
CVCL_W981	PER-278	9	75.00%	X,Y	11,12	23,25	18	12,13	11,12	11,12	8,9	13	14,15	13,15	29,31	24,27			6,8	8,11	14,19
CVCL_4641	JHOC-7	9	73.33%	X	11,12			9,12	11,12		8,10	9							9	8,11	14,17
CVCL_2979	KML-1	9	73.33%	X	11,12		16,17	9,12	11,12	13,15	8	11	13		29,32.2	20	11,12	17,19	7,9	8,11	16,18
CVCL_WR71	LaNCE hiPSC-16	9	73.33%	X,Y	10,11	19	14,17	12	11,12	11,16	10,12	12,13	12,14	13,14.2	30	23,24			6	8,11	15
CVCL_2661 *Best*	OE21	9	73.33%	X,Y	11,12	17,19	17	12,13	11,12	12	12	13	19	15.2	30,31	20	11,12	12	8	8,11	16,17
CVCL_2661 *Worst*	OE21	9	60.00%	X	11,12	17,19	17	13	11,12	12	12	12,13	19	15.2	30,31	20	11,12	12	7,8	8,11	15,16
CVCL_0181	BV-173	9	70.97%	X	11,12		16,17	10,12	10,11	11,12,13	8,10	11,13	12,16		30,32	20,24	11	12,16	6,9.3	8,10	16
CVCL_YP34	H58	9	70.97%	X,Y	12			12	11,12		9,11	11,13			28,30				6,9.3	8,11	16,17
CVCL_8403	PSVK1	9	70.97%	X,Y	11,12			12,13	10,11		12	9,13							6,7	8,11	16
CVCL_JE76	UCSF-OT-1109	9	70.97%	X	11,12			12	10,12		8,12	9,13							6,7	8,11	16,18
CVCL_WZ13	17978	9	68.97%	X	11,12	19,25	15,17	11,12	11	12	10	12,13	13,15	12,14	29,33.2	25	9,10	16,17	6,9.3	8	16,18
CVCL_1125 *Best*	CHP-212	9	68.97%	X,Y	12		15,17	10,12	11	11,13	8	13	14,16		27,29	20	9,10	5,15	6,7	8,11	15,18
CVCL_1125 *Worst*	CHP-212	9	68.97%	X,Y	12		15,17	10,12	11	11,13	8,13	13	14,16		27,29	20	9,10	5,15	6	8,11	15,18
CVCL_C609 *Best*	FM82	9	68.97%	X	14		18	12	11		8,10	11,13				24,25			6,8	8,11	15,16
CVCL_C609 *Worst*	FM82	9	64.29%	X	14		18	12	11		8	11,13				24,25			6,8	8,11	15,16
CVCL_9746	CHA-hES20	9	68.75%	X,Y	11,12		14,15	12,13	11,12	12,16	8,11	9	15,16		30,31	23,25	12	16,17	6,9	8,11	14,17
CVCL_A975	chHES-60	9	68.75%	X	11,12		15	9,10	11,12	13,16	8,12	11,13	14,15		29,31	20,24	12,13	14,16	6,7	8,11	17,18
