# Supplementary material for: PD‐L1 on Tumor‐Derived Extracellular Vesicles Induces CD8+ T Cell Terminal Exhaustion and Mediates Anti‐PD‐1 Resistance in Head and Neck Squamous Cell Carcinoma
Source: Adv Sci (Weinh). 2025 Nov 5;13(4):e16348. doi: 10.1002/advs.202516348 (PMC12822461; doi:10.1002/advs.202516348)
Supplement: Supplementary file 15 — Supporting Information [file ADVS-13-e16348-s010.docx]

**Supplementary Figures**

**
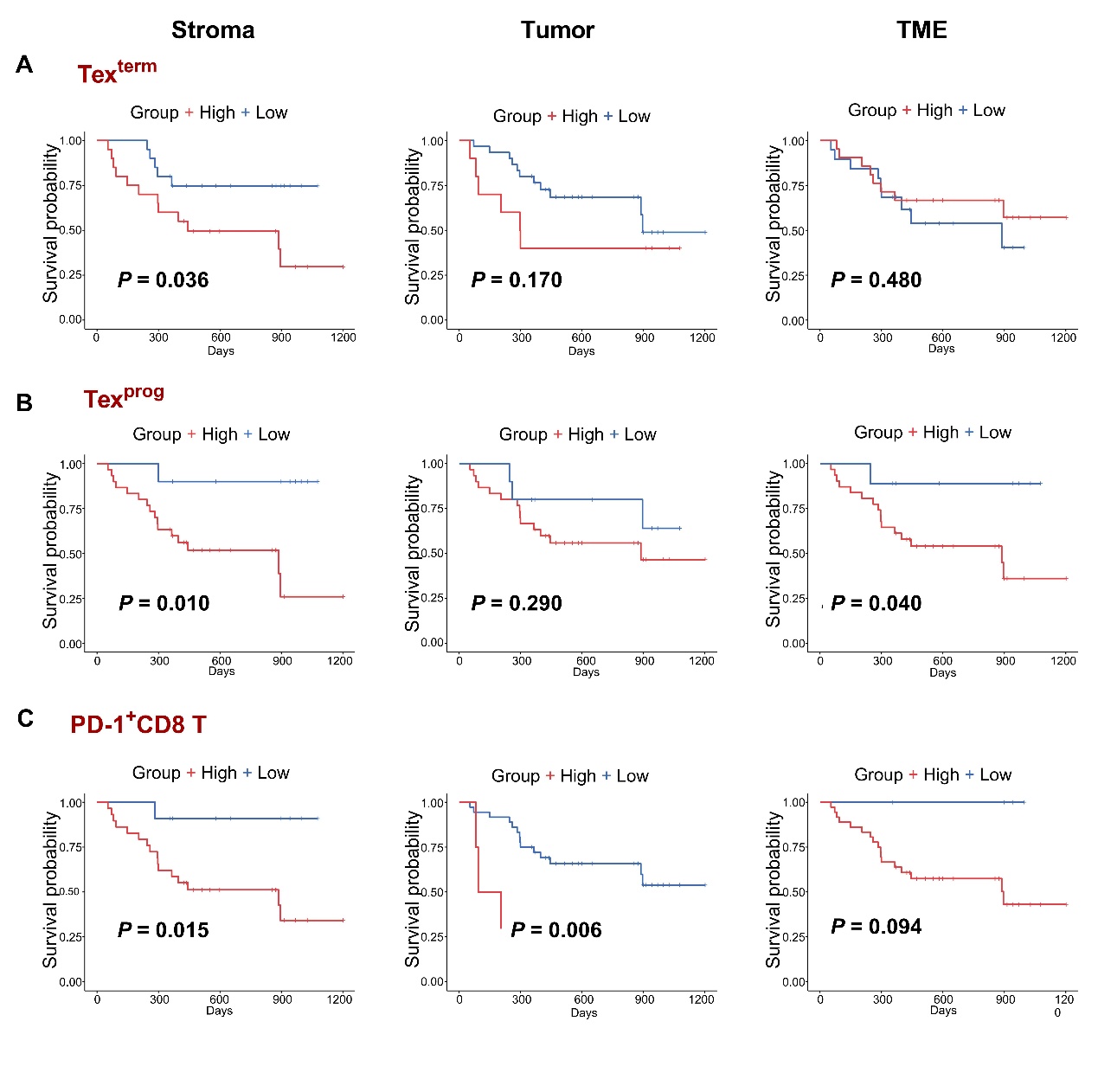
**

**Figure S1.** Kaplan-Meier curves depict the association between the density of CD8⁺ (A）terminal exhausted T (Tex^term^) cells; (B) progenitor exhausted T (Tex^prog^) cells and (C) PD-1^+^CD8 T cells within the stromal, tumor, and tumor microenvironment (TME) regions, and patient progression-free survival (PFS).


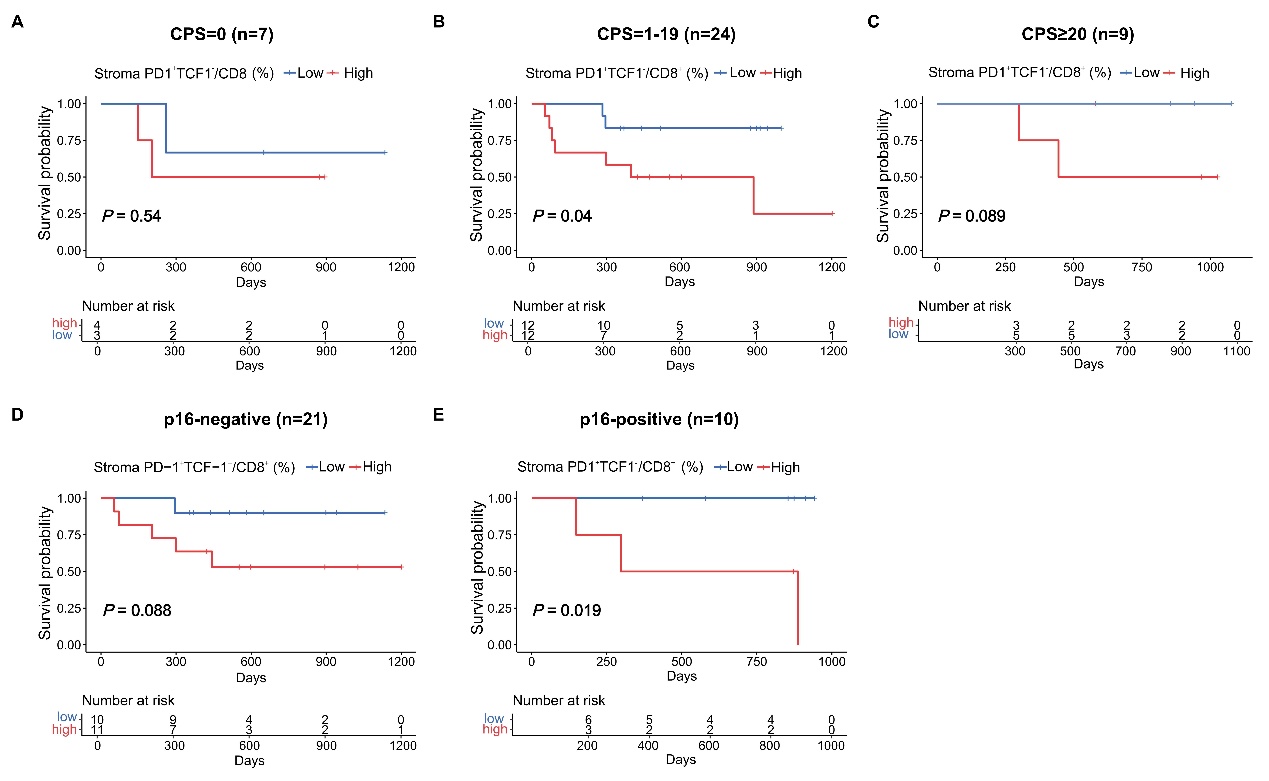


**Figure S2**. Kaplan–Meier analyzes of the subgroup of patients with CPS=0 (A), CPS=1-19 (B), CPS≥20 (C), p16-negative (D) and p16-positive (E) .

**Legends for Supplementary Tables**

**Table S1.** Differential Gene Expression of CD8^+^ cell subset (C1, C2, and C3) in the GSE234933 dataset, including key metrics such as gene markers, expression values, *P*-values, and adjusted p-values, aimed at identifying significant genes that distinguish between the different CD8^+^ cell subset.

**Table S2.** Cell identification along with their corresponding cluster assignments and original sample identifiers in the GSE234933 dataset.

**Table S3.** GO enrichment analysis for CD8-C2-Tex^term^ cell subset in the GSE234933 dataset, including GO IDs, terms, associated genes, descriptions, p-values, false discovery rates (FDR), and enrichment scores.

**Table S4.** Baseline characteristics and CD8^+^ T cell subset infiltration in tumor, stroma, and TME area of 40 HNSCC patients receiving aPD-1 treatment.

**Table S5.** Expression Levels of *CD274* (PD-L1) gene across tumor cells in the samples from the GSE234933 dataset.

**Table S6.** Expression levels of the *CD274* gene (PD-L1) in positively expressing cells across various cell subsets in samples from the GSE234933 dataset.

**Table S7.** All the differentially expressed genes in PD-L1^+^ tumors compared to PD-L1^-^ tumors (P < 0.05).

**Table S8.** Gene expression differences between the group of PD-L1^KO^ EVs and PD-L1^+^ EVs.

**Table S9.** Gene expression differences between the group of PD-L1^+^ EVs and PBS.

**Table S10.** GSEA enrichment analysis of the different expression genes (DEGs) between the group of PD-L1^+^ EVs and PD-L1^KO^ EVs.

**Table S11.** TCGA analysis identified prognosis-related genes in head and neck squamous cell carcinoma.

**Table S12.** Basic information of 34 patients with HNSCC .

**Table S13.** Basic information and treatment outcomes of 40 patients with HNSCC who received immunochemotherapy as the initial therapy.

**References:**

1.M. Tahara, R. Greil, and D. Rischin, et al., "659MO Pembrolizumab with or without chemotherapy for first-line treatment of recurrent/metastatic (R/M) head and neck squamous cell carcinoma (HNSCC): 5-year results from KEYNOTE-048," *Annals of Oncology* 33, no. null (2022): S844.

2.N. Colombo, C. Dubot, and D. Lorusso, et al., "Pembrolizumab for Persistent, Recurrent, or Metastatic Cervical Cancer.," *New England Journal of Medicine* 385, no. 20 (2021): 1856-1867.

3.J. M. Sun, L. Shen, and M. A. Shah, et al., "Pembrolizumab plus chemotherapy versus chemotherapy alone for first-line treatment of advanced oesophageal cancer (KEYNOTE-590): a randomised, placebo-controlled, phase 3 study.," *Lancet* 398, no. 10302 (2021): 759-771.

4.C. Robert, J. Schachter, and G. V. Long, et al., "Pembrolizumab versus Ipilimumab in Advanced Melanoma.," *New England Journal of Medicine* 372, no. 26 (2015): 2521-2532.

5.R. J. Motzer, N. M. Tannir, and D. F. McDermott, et al., "Nivolumab plus Ipilimumab versus Sunitinib in Advanced Renal-Cell Carcinoma.," *New England Journal of Medicine* 378, no. 14 (2018): 1277-1290.

6.V. Makker, N. Colombo, and A. Santin, et al., "Efficacy of next line of therapy after treatment with lenvatinib (LEN) in combination with pembrolizumab (pembro) versus treatment of physician’s choice (TPC) in patients (pts) with advanced endometrial cancer (aEC): Exploratory analysis of Study 309/KEYNOTE-775.," *Journal of Clinical Oncology* 40, no. 16_suppl (2022): 5587.

7.J. P. Machiels, Y. Tao, and L. Licitra, et al., "Pembrolizumab plus concurrent chemoradiotherapy versus placebo plus concurrent chemoradiotherapy in patients with locally advanced squamous cell carcinoma of the head and neck (KEYNOTE-412): a randomised, double-blind, phase 3 trial.," *Lancet Oncology* 25, no. 5 (2024): 572-587.

8.B. Burtness, K. J. Harrington, and R. Greil, et al., "Pembrolizumab alone or with chemotherapy versus cetuximab with chemotherapy for recurrent or metastatic squamous cell carcinoma of the head and neck (KEYNOTE-048): a randomised, open-label, phase 3 study," *Lancet* 394, (2019):1915-1928.

9.R. L. Ferris, G. J. Blumenschein, and J. Fayette, et al., "Nivolumab for Recurrent Squamous-Cell Carcinoma of the Head and Neck," *New England Journal of Medicine* 375, (2016): 1856-1867.

10.Z. Cai, L. Chen, and S. Chen, et al., "Single-cell RNA sequencing reveals pro-invasive cancer-associated fibroblasts in hypopharyngeal squamous cell carcinoma.," *Cell Communication and Signaling* 21, no. 1 (2023): 292.

11.F. Wei, R. Fang, and K. Lyu, et al., "Exosomal PD-L1 derived from head and neck squamous cell carcinoma promotes immune evasion by activating the positive feedback loop of activated regulatory T cell-M2 macrophage.," *Oral Oncology* 145, no. null (2023): 106532.

12.M. Philip and A. Schietinger, "CD8(+) T cell differentiation and dysfunction in cancer," *Nature Reviews Immunology* 22, (2022): 209-223.

13.S. J. Im, M. Hashimoto, and M. Y. Gerner, et al., "Defining CD8+ T cells that provide the proliferative burst after PD-1 therapy," *Nature* 537, (2016): 417-421.

14.R. He, S. Hou, and C. Liu, et al., "Follicular CXCR5- expressing CD8(+) T cells curtail chronic viral infection.," *Nature* 537, (2016): 412-428.

15.W. Chen, J. Teo, and S. W. Yau, et al., "Chronic type I interferon signaling promotes lipid-peroxidation-driven terminal CD8+ T cell exhaustion and curtails anti-PD-1 efficacy.," *Cell Reports* 41, no. 7 (2022): 111647.

16.Y. Guo, Y. Q. Xie, and M. Gao, et al., "Metabolic reprogramming of terminally exhausted CD8+ T cells by IL-10 enhances anti-tumor immunity.," *Nature Immunology* 22, no. 6 (2021): 746-756.

17.J. C. Beltra, S. Manne, and M. S. Abdel-Hakeem, et al., "Developmental Relationships of Four Exhausted CD8+ T Cell Subsets Reveals Underlying Transcriptional and Epigenetic Landscape Control Mechanisms.," *Immunity* 52, no. 5 (2020): 825-841.

18.B. Liu, X. Hu, and K. Feng, et al., "Temporal single-cell tracing reveals clonal revival and expansion of precursor exhausted T cells during anti-PD-1 therapy in lung cancer," *Nature Cancer*. 3, 108-121.(2022).

19.L. Zheng, S. Qin, and W. Si, et al., "Pan-cancer single-cell landscape of tumor-infiltrating T cells," *Science* 374, (2021): abe6474.

20.J. S. Dolina, N. Van Braeckel-Budimir, G. D. Thomas, and S. Salek-Ardakani, "CD8(+) T Cell Exhaustion in Cancer," *Frontiers in Immunology* 12, (2021): 715234.

21.F. Franco, A. Jaccard, P. Romero, Y. R. Yu, and P. C. Ho, "Metabolic and epigenetic regulation of T-cell exhaustion," *Nature Metabolism* 2, (2020): 1001-1012.

22.P. Vignali, K. DePeaux, and M. Watson, et al., "512 Terminally exhausted CD8 T cells potentiate the tolerogenic tumor microenvironment as functional suppressors," *Journal for Immunotherapy of Cancer* 8, no. Suppl 3 (2020): A548.

23.B. Diskin, S. Adam, and M. F. Cassini, et al., "PD-L1 engagement on T cells promotes self-tolerance and suppression of neighboring macrophages and effector T cells in cancer.," *Nature Immunology* 21, no. 4 (2020): 442-454.

24.E. D. Lucas, J. B. Schafer, and J. Matsuda, et al., "PD-L1 Reverse Signaling in Dermal Dendritic Cells Promotes Dendritic Cell Migration Required for Skin Immunity.," *Cell Reports* 33, no. 2 (2020): 108258.

25.A. Kornepati, R. K. Vadlamudi, and T. J. Curiel, "Programmed death ligand 1 signals in cancer cells.," *Nature Reviews Cancer* 22, no. 3 (2022): 174-189.

26.F. Wei, R. Fang, and K. Lyu, et al., "Exosomal PD-L1 derived from head and neck squamous cell carcinoma promotes immune evasion by activating the positive feedback loop of activated regulatory T cell-M2 macrophage," *Oral Oncology* 145, (2023): 106532.

27.Y. Chu, E. Dai, and Y. Li, et al., "Pan-cancer T cell atlas links a cellular stress response state to immunotherapy resistance.," *Nature Medicine* 29, (2023): 1550-1562.

28.F. Bray, M. Laversanne, and H. Sung, et al., "Global cancer statistics 2022: GLOBOCAN estimates of incidence and mortality worldwide for 36 cancers in 185 countries.," *Ca-a Cancer Journal for Clinicians* 74, no. 3 (2024): 229-263.

29.E. S. Ch'Ng, "Head and Neck Cancer.," *New England Journal of Medicine* 382, no. 20 (2020): e57.

30.B. Burtness, K. J. Harrington, and R. Greil, et al., "Pembrolizumab alone or with chemotherapy versus cetuximab with chemotherapy for recurrent or metastatic squamous cell carcinoma of the head and neck (KEYNOTE-048): a randomised, open-label, phase 3 study.," *Lancet* 394, no. 10212 (2019): 1915-1928.

31.S. J. Im, M. Hashimoto, and M. Y. Gerner, et al., "Defining CD8+ T cells that provide the proliferative burst after PD-1 therapy.," *Nature* 537, (2016): 417-421.

32.J. C. Beltra, S. Manne, and M. S. Abdel-Hakeem, et al., "Developmental Relationships of Four Exhausted CD8(+) T Cell Subsets Reveals Underlying Transcriptional and Epigenetic Landscape Control Mechanisms," *Immunity* 52, (2020): 825-841.

33.C. U. Blank, W. N. Haining, and W. Held, et al., "Defining 'T cell exhaustion'.," *Nature Reviews Immunology* 19, no. 11 (2019): 665-674.

34.P. Zhou, H. Shi, and H. Huang, et al., "Single-cell CRISPR screens in vivo map T cell fate regulomes in cancer.," *Nature* 624, (2023): 154-163.

35.B. C. Miller, D. R. Sen, and R. Al Abosy, et al., "Subsets of exhausted CD8+ T cells differentially mediate tumor control and respond to checkpoint blockade.," *Nature Immunology* 20, no. 3 (2019): 326-336.

36.I. Siddiqui, K. Schaeuble, and V. Chennupati, et al., "Intratumoral Tcf1(+)PD-1(+)CD8(+) T Cells with Stem-like Properties Promote Tumor Control in Response to Vaccination and Checkpoint Blockade Immunotherapy," *Immunity* 50, (2019): 195-211.

37.S. Koyama, E. A. Akbay, and Y. Y. Li, et al., "Adaptive resistance to therapeutic PD-1 blockade is associated with upregulation of alternative immune checkpoints.," *Nature Communications* 7, no. null (2016): 10501.

38.Null, "Exhausted T cells in tumors gain suppressor activity and can restrain immunity.," *Nature Immunology* 24, no. 2 (2023): 218-219.

39.D. Wang, J. Fang, and S. Wen, et al., "A comprehensive profile of TCF1+ progenitor and TCF1- terminally exhausted PD-1+CD8+ T cells in head and neck squamous cell carcinoma: implications for prognosis and immunotherapy.," *International Journal of Oral Science* 14, no. 1 (2022): 8.

40.P. Falvo, S. Orecchioni, and R. Hillje, et al., "Abstract 1653: A single-cell atlas of the effect of chemotherapeutics over intratumoral immune cells reveals that combining an alkylating agent and a vinca alkaloid can activate antigen presenting cells and increase tcf1 stem-like CD8 T-cells, thus improving anti-PD-1 efficacy in triple negative breast cancer and lymphoma," *Cancer Research* 81, no. 13_Supple (2021): 1653.

41.D. B. Doroshow, S. Bhalla, and M. B. Beasley, et al., "PD-L1 as a biomarker of response to immune-checkpoint inhibitors," *Nature Reviews Clinical Oncology* 18, (2021): 345-362.

42.R. Fang, Y. Chen, and B. Huang, et al., "Predicting response to PD-1 inhibitors in head and neck squamous cell carcinomas using peripheral blood inflammatory markers.," *Translational Oncology* 51, (2025): 102222.

43.J. H. Strickler, B. A. Hanks, and M. Khasraw, "Tumor Mutational Burden as a Predictor of Immunotherapy Response: Is More Always Better?" *Clinical Cancer Research* 27, (2021): 1236-1241.

44.I. Siddiqui, K. Schaeuble, and V. Chennupati, et al., "Intratumoral Tcf1+PD-1+CD8+ T Cells with Stem-like Properties Promote Tumor Control in Response to Vaccination and Checkpoint Blockade Immunotherapy.," *Immunity* 50, no. 1 (2019): 195-211.

45.J. Waibl Polania, A. Hoyt-Miggelbrink, and W. H. Tomaszewski, et al., "Antigen presentation by tumor-associated macrophages drives T cells from a progenitor exhaustion state to terminal exhaustion.," *Immunity* 58, (2025): 232-246.

46.J. Beltra, S. Manne, and M. Hakeem, et al., "Developmental relationships of four exhausted CD8 T cell subsets reveals underlying transcriptional and epigenetic control mechanisms," *Journal of Immunology* 204, no. 1_Supple (2020): 16-77.

47.Y. Fan, X. Che, and J. Qu, et al., "Exosomal PD-L1 Retains Immunosuppressive Activity and is Associated with Gastric Cancer Prognosis," *Annals of Surgical Oncology* 26,(2019): 3745-3755.

48.G. Chen, A. C. Huang, and W. Zhang, et al., "Exosomal PD-L1 contributes to immunosuppression and is associated with anti-PD-1 response.," *Nature* 560, no. 7718 (2018): 382-386.

49.M. N. Theodoraki, S. S. Yerneni, T. K. Hoffmann, W. E. Gooding, and T. L. Whiteside, "Clinical Significance of PD-L1(+) Exosomes in Plasma of Head and Neck Cancer Patients," *Clinical Cancer Research* 24, (2018): 896-905.

50.C. Yao, H. W. Sun, and N. E. Lacey, et al., "Single-cell RNA-seq reveals TOX as a key regulator of CD8+ T cell persistence in chronic infection.," *Nature Immunology* 20, no. 7 (2019): 890-901.

51.O. Khan, J. R. Giles, and S. McDonald, et al., "TOX transcriptionally and epigenetically programs CD8+ T cell exhaustion.," *Nature* 571, (2019): 211-218.

52.L. M. McLane, S. F. Ngiow, and Z. Chen, et al., "Role of nuclear localization in the regulation and function of T-bet and Eomes in exhausted CD8 T cells.," *Cell Reports* 35, no. 6 (2021): 109120.

53.X. Liu, Y. Wang, and H. Lu, et al., "Genome-wide analysis identifies NR4A1 as a key mediator of T cell dysfunction.," *Nature* 567, no. 7749 (2019): 525-529.

54.H. Seo, J. Chen, and E. González-Avalos, et al., "TOX and TOX2 transcription factors cooperate with NR4A transcription factors to impose CD8+ T cell exhaustion.," *Proceedings of the National Academy of Sciences of the United States of America* 116, no. 25 (2019): 12410-12415.

55.S. K. Boi, X. Lan, and B. Youngblood, "BATF targets T cell exhaustion for termination.," *Nature Immunology* 22, no. 8 (2021): 936-938.

56.F. Alfei, K. Kanev, and M. Hofmann, et al., "TOX reinforces the phenotype and longevity of exhausted T cells in chronic viral infection.," *Nature* 571, (2019): 265-269.

57.B. Wu, A. N. Koehler, and P. Westcott, "New opportunities to overcome T cell dysfunction: the role of transcription factors and how to target them.," *Trends in Biochemical Sciences* 49, no. 11 (2024): 1014-1029.

58.V. A. Boussiotis, "Molecular and Biochemical Aspects of the PD-1 Checkpoint Pathway.," *New England Journal of Medicine* 375, no. 18 (2016): 1767-1778.

59.M. Quigley, F. Pereyra, and B. Nilsson, et al., "Integrative Genomic Analysis of HIV-Specific CD8 T Cells Reveals That PD-1 Inhibits T Cell Function by Upregulating the AP-1 Transcription Factor BATF.," *Blood* 114, no. 22 (2009): 916.

60.S. Sui, M. Zhong, and S. Zhong, et al., "BRD4 inhibitor reduces exhaustion and blocks terminal differentiation in CAR-T cells by modulating BATF and EGR1.," *Biomarker Research* 12, (2024): 124.

61.C. Théry, S. Amigorena, G. Raposo, and A. Clayton, "Isolation and characterization of exosomes from cell culture supernatants and biological fluids.," Curr Protoc Cell Biol. Chapter 3, 3-22.(2006).

62.Y. Li, Z. K. Chen, and X. Duan, et al., "Targeted inhibition of tumor-derived exosomes as a novel therapeutic option for cancer.," *Experimental and Molecular Medicine* 54, no. 9 (2022): 1379-1389.

63.J. H. Kim, C. H. Lee, and M. C. Baek, "Dissecting exosome inhibitors: therapeutic insights into small-molecule chemicals against cancer.," *Experimental and Molecular Medicine* 54, no. 11 (2022): 1833-1843.

64.K. Shahraki, P. G. Boroumand, and H. Lotfi, et al., "An update in the applications of exosomes in cancer theranostics: from research to clinical trials.," *Journal of Cancer Research and Clinical Oncology* 149, no. 10 (2023): 8087-8116.

65.R. Surana, V. LeBleu, and J. Lee, et al., "Phase I study of mesenchymal stem cell (MSC)-derived exosomes with KRAS^G12D^ siRNA in patients with metastatic pancreatic cancer harboring a KRAS^G12D^ mutation.," *Journal of Clinical Oncology* 40, no. 4_suppl (2022): TPS633.

66.B. Baek, M. Kim, and J. Kim, et al., "Abstract 2719: Papiliximab, a bispecific nanobody targeting CD47 and PDL1 retards tumor growth without hemolysis," *Cancer Research* 84, no. 6_Supple (2024): 2719.

67.Z. Li, Z. Chen, and K. Shi, et al., "Polyphenol-Based Self-Assembled Nanomedicine for a Three-Pronged Approach to Reversing Tumor Immunosuppression.," *Advanced Healthcare Materials* -, (2024): e2402127.

68.R. Bill, P. Wirapati, and M. Messemaker, et al., "CXCL9:SPP1 macrophage polarity identifies a network of cellular programs that control human cancers," *Science* 381,(2023): 515-524.

69.A. Obradovic, D. Graves, and M. Korrer, et al., "Immunostimulatory Cancer-Associated Fibroblast Subpopulations Can Predict Immunotherapy Response in Head and Neck Cancer," *Clinical Cancer Research* 28, (2022): 2094-2109.

70.A. M. Luoma, S. Suo, and Y. Wang, et al., "Tissue-resident memory and circulating T cells are early responders to pre-surgical cancer immunotherapy," *Cell* 185, (2022): 2918-2935.

71.S. Chen, Y. Zhou, Y. Chen, and J. Gu, "fastp: an ultra-fast all-in-one FASTQ preprocessor," *Bioinformatics* 34, (2018): i884-i890.

72.T. Stuart, A. Butler, and P. Hoffman, et al., "Comprehensive Integration of Single-Cell Data," *Cell*. 177, (2019): 1888-1902.

73.R. Vento-Tormo, M. Efremova, and R. A. Botting, et al., "Single-cell reconstruction of the early maternal-fetal interface in humans.," *Nature* 563, no. 7731 (2018): 347-353.

74.K. Ito and D. Murphy, "Application of ggplot2 to Pharmacometric Graphics," *Cpt-Pharmacometrics & Systems Pharmacology* 2, (2013): e79.

75.S. Ruan, M. Lin, and Y. Zhu, et al., "Integrin beta4-Targeted Cancer Immunotherapies Inhibit Tumor Growth and Decrease Metastasis," *Cancer Research* 80, (2020): 771-783.

76.C. Tsui, L. Kretschmer, and S. Rapelius, et al., "MYB orchestrates T cell exhaustion and response to checkpoint inhibition," *Nature* 609, (2022): 354-360.

77.R. Ford, P. Vignali, and N. Rittenhouse, et al., "518 Epigenetic dysfunction of terminally exhausted tumor infiltrating T cells," *Journal for Immunotherapy of Cancer* 8, no. Suppl 3 (2020): A554.

78.M. Brand, S. Laban, and M. N. Theodoraki, et al., "Characterization and Differentiation of the Tumor Microenvironment (TME) of Orthotopic and Subcutaneously Grown Head and Neck Squamous Cell Carcinoma (HNSCC) in Immunocompetent Mice," *International Journal of Molecular Sciences* 22, (2020).
